# Coiled-coil inspired functional inclusion bodies

**DOI:** 10.1186/s12934-020-01375-4

**Published:** 2020-06-01

**Authors:** Marcos Gil-Garcia, Susanna Navarro, Salvador Ventura

**Affiliations:** Institut de Biotecnologia i de Biomedicina and Departament de Bioquímica i Biologia Molecular, Universitat Autònoma de Barcelona, 08193 Bellaterra, Barcelona Spain

**Keywords:** Coiled-coil protein, Fusion tag, Functional inclusion bodies, Fluorescent proteins, Protein engineering

## Abstract

**Background:**

Recombinant protein expression in bacteria often leads to the formation of intracellular insoluble protein deposits, a major bottleneck for the production of soluble and active products. However, in recent years, these bacterial protein aggregates, commonly known as inclusion bodies (IBs), have been shown to be a source of stable and active protein for biotechnological and biomedical applications. The formation of these functional IBs is usually facilitated by the fusion of aggregation-prone peptides or proteins to the protein of interest, leading to the formation of amyloid-like nanostructures, where the functional protein is embedded.

**Results:**

In order to offer an alternative to the classical amyloid-like IBs, here we develop functional IBs exploiting the coiled-coil fold. An in silico analysis of coiled-coil and aggregation propensities, net charge, and hydropathicity of different potential tags identified the natural homo-dimeric and anti-parallel coiled-coil ZapB bacterial protein as an optimal candidate to form assemblies in which the native state of the fused protein is preserved. The protein itself forms supramolecular fibrillar networks exhibiting only α-helix secondary structure. This non-amyloid self-assembly propensity allows generating innocuous IBs in which the recombinant protein of interest remains folded and functional, as demonstrated using two different fluorescent proteins.

**Conclusions:**

Here, we present a proof of concept for the use of a natural coiled-coil domain as a versatile tool for the production of functional IBs in bacteria. This α-helix-based strategy excludes any potential toxicity drawback that might arise from the amyloid nature of β-sheet-based IBs and renders highly active and homogeneous submicrometric particles.

## Background

Biotechnological and pharmaceutical industries exploit microorganisms as cell factories in order to produce their biological products, including therapeutic proteins, such as hormones, enzymes for replacement therapies, or antibodies [1, 2]. However, the production of these molecules in their soluble and functional states faces significant barriers [3, 4]. Proteins have been shaped by natural selection to remain soluble and functional under physiological conditions, according to the “living on the edge” hypothesis [5]. The heterologous expression of these molecules in bacteria leads to intracellular concentrations that are several times above their natural solubility limits. As a result, these proteins might establish non-native inter-molecular interactions, which would facilitate their aggregation into inclusion bodies (IBs) in the bacterial cytosol [6].

Traditionally IBs were thought to be formed by misfolded conformations and thus devoid of any functionality. However, data is accumulating to indicate that, at least for specific proteins and production conditions, IBs might exhibit significant activity [7, 8]. IBs are easy to purify and can be stored for long periods, thanks to their inherent stability [9]. They have a nanometric size (50–1000 nm) [9–12], and ~ 90% of them are composed of the target protein [13–15]. These properties, combined, make them active nanoparticles, which are finding increasing applications in biotechnology and biomedicine. In this way, the ability to immobilize enzymes in IBs has been exploited to build up reusable catalysts [16, 17], and IBs have been used as nanocarriers or/and nanopills to deliver antitumoral polypeptides in the body [18, 19].

We have shown that, generically, a significant proportion of the protein contacts that sustain IBs have an amyloid-like nature [20–22]. When embedded in IBs, proteins exhibit a significant increase in β-sheet content, relative to their soluble counterparts, and, often, become able to bind typical amyloid dyes. For specific proteins or protein fusions, this amyloid scaffold coexists with functional conformations [23, 24]. However, because the process of protein aggregation into non-native intermolecular β-sheet structures necessarily involves the population of misfolded species, a fraction of the recombinant protein is necessarily inactivated to build up the amyloid structure that sustains the IB [22].

IBs have been assimilated to natural functional amyloids [25], which are non-toxic for their host cells or organisms [26–28]. However, different studies indicate that this is not because functional amyloids are intrinsically non-cytotoxic [29], but instead because, in these specific cases, nature has evolved dedicated mechanisms to prevent amyloid-associated toxicity [27, 30, 31]. The artificial formation of IBs lack these natural control mechanisms and, although bacterial aggregates have been assumed to be innocuous, it cannot be entirely discarded that toxic β-sheet conformations can be incorporated or released from these amyloid-like inclusions.

In order to overcome the two above-described limitations, we introduce here an IBs production strategy that exploits a natural coiled-coil protein to promote non-amyloid supramolecular interactions. In the last years, the use and design of coiled-coil domains as building blocks in protein assemblies have attracted significant attention [32, 33]. The self-organization capacity of these structures has been exploited to create different nanostructures, such as nanofibers [34] and nanocages [35, 36]. Furthermore, two different coiled-coil domains have been previously used to generate active IBs: the tetramerization domain of the tetrabrachion protein (TDoT) from *Staphylothermus marinus* [16, 37–40] and the 3HAMP coiled-coil, which was derived from the oxygen sensor protein Aer2 from *Pseudomonas aeruginosa* [37, 41]. In this work, we apply this strategy to build up functional IBs using ZapB, a non-essential *Escherichia coli* (*E. coli*) protein consisting of two anti-parallel α-helices, involved in Z-ring formation during the bacterial cell division process [42, 43].

We first show that the ZapB self-assembles to form α-helix-based fibrillar networks, and afterward, we demonstrate how this property allows its use as a tag to form non-amyloid and non-toxic IBs which preserve the activity of the attached polypeptides.

## Results and discussion

### Selection of a polar and non-aggregating coiled-coil protein for the production of functional IBs

A wide range of fusion tags has been used to induce IBs formation. They comprise small artificial peptides [44], and aggregation-prone natural proteins or domains [22, 37, 45], which are fused to functional globular proteins. A characteristic property of most of these tags is that they promote the formation of aggregates sustained by collective intermolecular β-sheet interactions. Alternatively, two different coiled-coil domains have been used for the production of fluorescent and/or catalytically IBs [37]. Nevertheless, the biophysical properties of these active aggregates were not assessed, and thus it is not known if the coiled-coil encoding sequences keep their native helical structure in the IBs or they had just transitioned to a conventional amyloid-like assembly. Indeed, the formation of β-sheet-rich amyloid fibrils by aggregation-prone coiled-coil sequences is behind the onset of several neurodegenerative disorders [46]. A similar transition in coiled-coil-tagged proteins during IBs formation might turn these aggregates potentially toxic.

We selected the *E. coli* protein ZapB as a scaffold to obtain functional IBs. ZapB is an 81 residues-long protein whose 3D-structure (PDB: 2JEE) consists of two α-helical polypeptide chains arranged in anti-parallel orientation to form a dimeric coiled-coil of 116 Å (PDB: 2JEE) [42]. In the crystal structure, individual coiled-coils interact close to their termini, which already suggested that, under appropriate conditions, these helical modules might self-assemble into supramolecular structures [42].

**Fig. 1:**
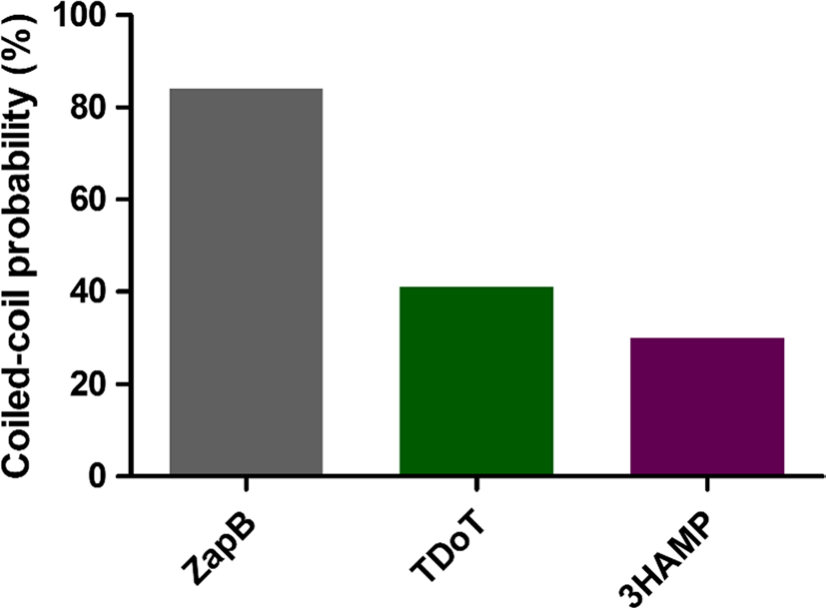
Coiled-coil probability for ZapB, TDoT, and 3HAMP. The coiled-coil propensity is represented according to DeepCoil predictor

The propensity to form a stable coiled-coil assembly in solution is encoded in the protein sequence. The higher the coiled-coil propensity, the lowest the probability to transition into an aggregated β-sheet structure since stable α-helices protect against aggregation [47, 48]. We calculated the coiled-coil propensity of ZapB and compared it with that of the two coiled-coil domains used as IBs formation tags in previous studies (3HAMP and TDoT) using four different algorithms: COILS [49], PCoils [50], MARCOIL [51] and DeepCoil [52]. Additional file 1: Figures S1–S3 show the coiled-coil probability profiles for ZapB, 3HAMP and TDoT. The four algorithms coincide to predict a very high coiled-coil propensity along the complete ZapB sequence. In the case of 3HAMP, the programs identify a region of high propensity close to the N-terminus and two additional stretches with low to moderate propensity. This is consistent with the homo-dimeric 3HAMP structure, in which parallel monomers exhibit three successive domains (HAMP1, 2, and 3), each about 50 residues long and bridged by flexible linkers. For TDoT, only DeepCoil is able to identify a significant coiled-coil propensity in the central part of the sequence. This makes sense, since TDoT is a parallel and right-handed coiled-coil tetramer, which is based on the 11-residue repeat, and COILS, PCoils and MARCOIL were trained to identify canonical heptad repeats, where DeepCoil was aimed to identify both kinds of periodicities. Therefore, we used this last algorithm to compare the average coiled-coil probabilities of ZapB, TDoT and 3HAMP primary sequences. As it can be seen in Fig. 1 ZapB seems to be a better coiled-coil former than the proteins it has been compared to.

**Fig. 2:**
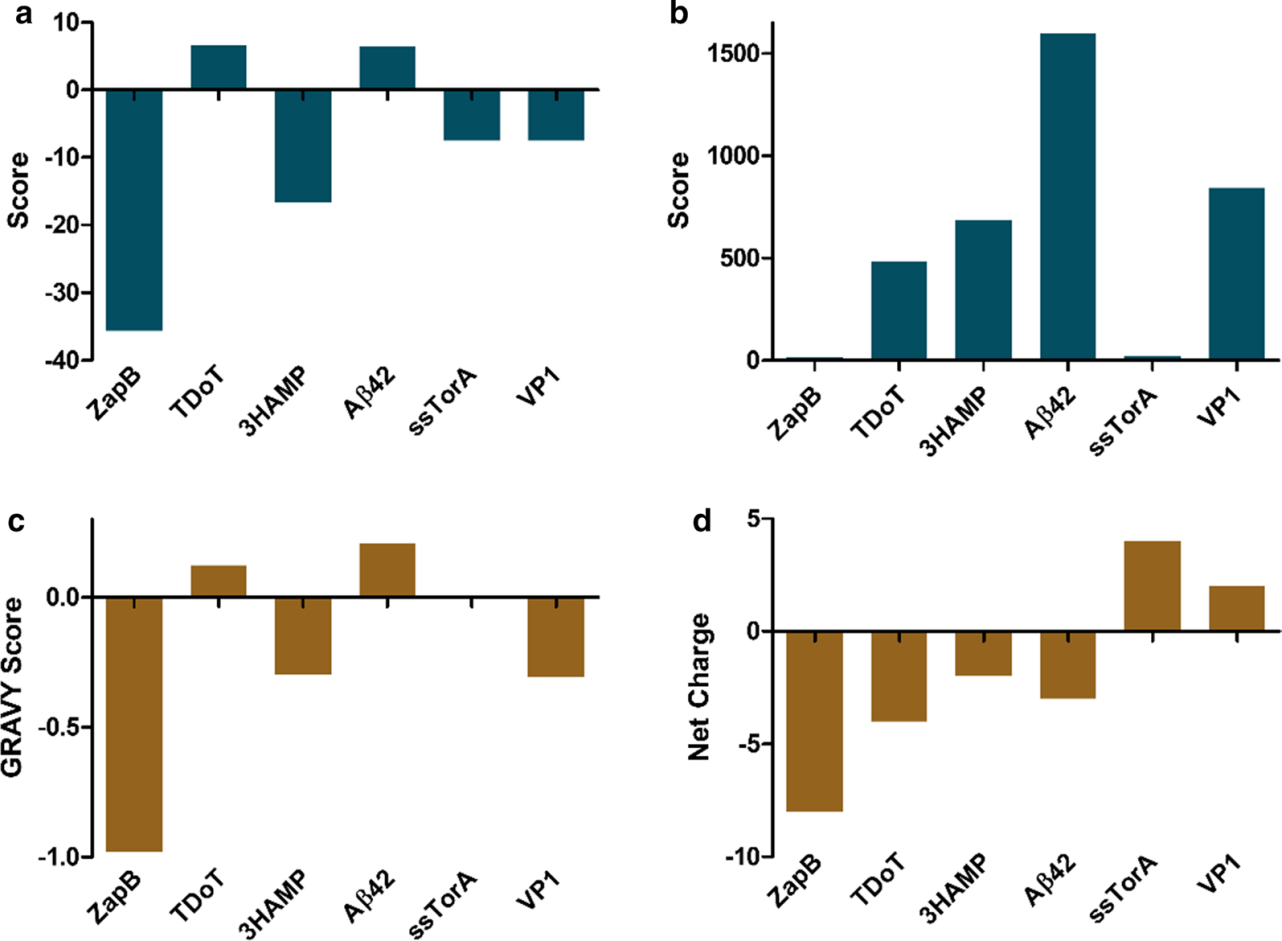
Aggregation propensity predictions, hydrophaticity scores, and net charge values. Aggregation propensity values calculated with **a** Aggrescan (Na4vSS value) and **b** TANGO (*AGG* value) for the six selected tags. For both predictors, the more positive the value, the higher the aggregation propensity. **c** GRAVY hydrophaticity score of the different tags according to the Kyte-Doolittle scale. **d** The calculated net charge of the sequences at physiological pH

Not only the coiled-coil propensity accounts for the ability to maintain the native state in the multimeric state, but also the protein’s intrinsic aggregation propensity, which facilitates the conformational shift to aggregated β-sheet states. We analyzed this property for ZapB, TDoT, 3HAMP, and other three non-coiled-coil sequences used previously as IB-tags, namely the amyloid β-peptide (Aβ42) [53, 54], the viral capsid peptide VP1 [45, 55], and the signal sequence of *E. coli* TorA (ssTorA) [56, 57]. In order to do that, we used two of the most popular sequence-based aggregation prediction servers, Aggrescan [58] and TANGO [59]. As it can be seen in Fig. 2a, b, ZapB was predicted as the least aggregation-prone sequence in this polypeptide set. Aggrescan (Fig. 2a), predicts ZapB to be the most soluble sequence with a significant difference, relative to the other proteins or domains. In the case of TANGO (Fig. 2b), ZapB remains as the less aggregation-prone sequence, in this case, together with ssTorA.

Polarity and hydrophobicity are critical negative and positive contributors to protein sequence aggregation propensity, respectively. Their role is crucial at the nucleation step of the aggregation reaction [60, 61]. These biophysical properties were analyzed by calculating the Grand Average of Hydropathicity (GRAVY score) provided by the ProtParam server, according to the Kyte-Doolittle scale [62]. Positive GRAVY scores indicate higher hydrophobicity, whereas negative values correspond to polar sequences. As it is shown in Fig. 2c, ZapB has the more negative GRAVY score (− 0.980), being this value three times higher than the secondly ranked sequence (VP1, GRAVY score = − 0.307), thus indicating that ZapB is a highly polar protein, relative to compared sequences.

We also checked the secondary structure propensity of the six tags. Cryptic regions of significant β-sheet propensity might exist even in sequences that usually fold into α-helices [63]. These stretches might favor aggregation into amyloid-like structures upon coiled-coil unfolding or once the polypeptide chain emerges from the ribosome [64]. To this aim, we used the PSIPRED [65] and GOR [66] servers. ZapB is predicted to be completely α-helix, with an extremely high propensity for this secondary structure; meanwhile, 3HAMP and specially TDoT are predicted to have a significantly lower α-helical propensity and predicted β-strand segments are identified at their sequences (Additional file 1: Figures S4, S5).

The above-described analyses converge to indicate that the sequence of ZapB is more polar, less aggregation-prone, devoid of cryptic β-sheet regions, and with highest α-helical and coiled-coil propensities than any previously used IBs tag. Each of these individual properties disfavors the potential aggregation of the ZapB sequence into β-sheet-rich aggregates. However, aggregation can still occur from the folded state of the proteins [67]. We have recently developed AGGRESCAN3D, an algorithm that allows predicting a protein’s aggregation-propensity taking into account the structural context [68–70]. When we analyzed the published 3D structures of the three coiled-coil domains (ZapB, PDB:2JEE; TDoT, PDB:1FE6; and 3HAMP, PDB:3LNR), ZapB turned to be the less aggregation-prone structure, displaying a highly soluble surface (Additional file 1: Figure S6).

Finally, we assessed the net charge of the different IBs tagging sequences. This value is essential because if the high predicted solubility of ZapB comes together with a high positive charge, this will be a significant drawback for its use in nanomedical applications. Cationic sequences bind to negatively charged nucleic acids and cell membranes, which then become incorporated into IBs during their formation or purification, making them compositionally heterogeneous. The calculated net charge for the six tags is represented in Fig. 2d. ZapB is the most acidic of the sequences (net charge − 8), the net charge being two times higher than that of the second most anionic tag (TDoT, net charge − 4). These tags differ in their lengths. In order to obtain a value independent of the protein size, we calculated the net charge per residue (NCPR) (Additional file 1: Figure S7) [71]. According to the NCPR values, ZapB is again the most acidic tag (NCPR: − 0.099), followed by TDoT (NCPR: − 0.077). Therefore, no interaction is expected between DNA, RNA, or membranes and ZapB.

Overall, we can conclude that, theoretically, ZapB fulfills all the requirements to work as a tag to promote the formation of non-amyloid-like functional IBs; still, to act as such, the domain should be able to self-assemble, despite its high predicted solubility in both the folded and unfolded states.

### ZapB self-assembles into coiled-coil, non-amyloid, nanofibers

**Fig. 3:**
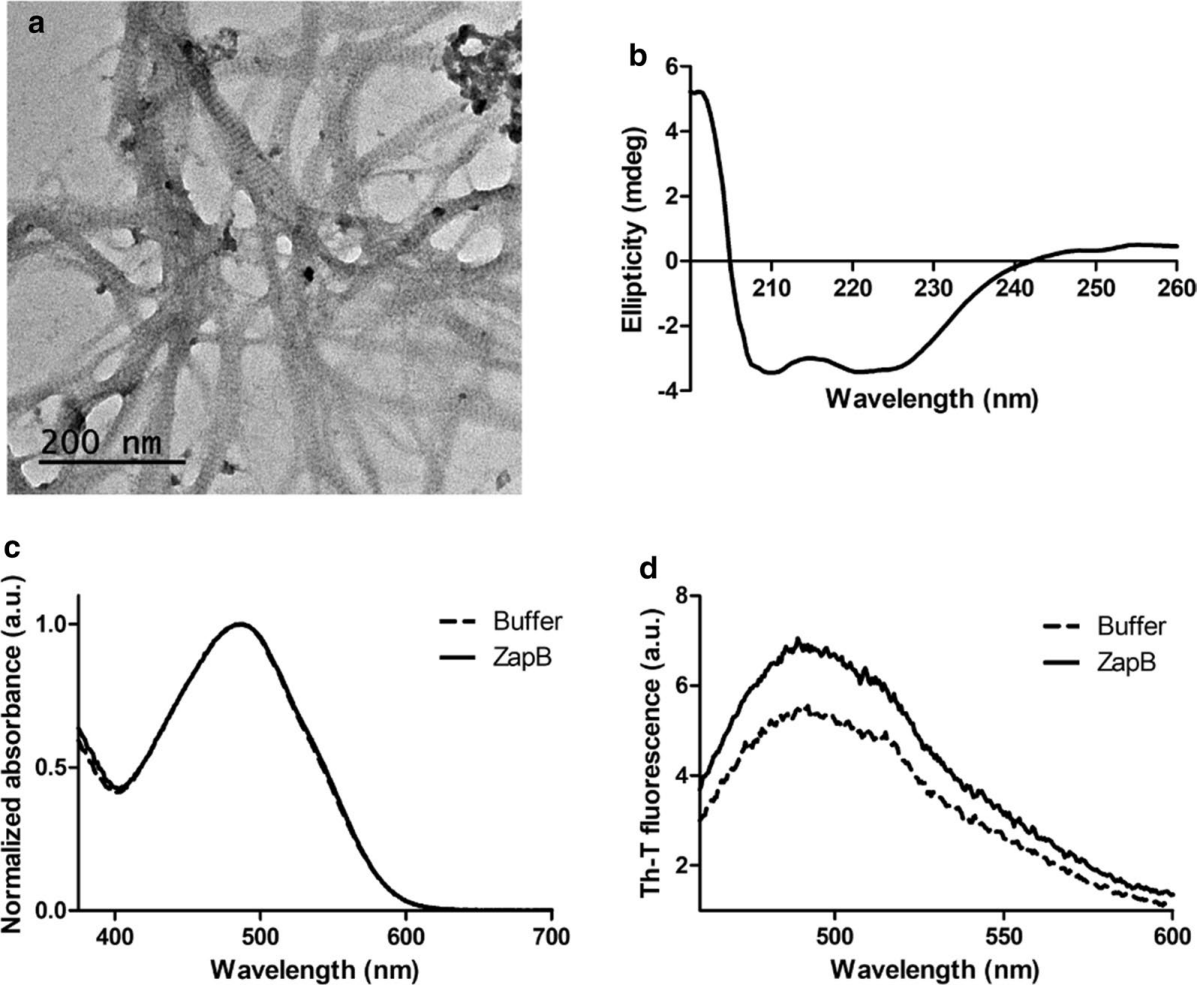
ZapB coiled-coil assembly biophysical characterization. **a** TEM image of ZapB fibers upon negative staining. **b** Far-UV circular dichroism spectrum. **c** Congo-Red absorbance spectra and **d** Th-T fluorescence emission spectra

In order to test the ability of ZapB to form protein assemblies driven by interactions between natively folded coiled-coils, a His-tag was added at the C-terminus of the full-length protein, and it was recombinantly produced in *E.coli* at 30 °C. The protein was expressed at high yield (> 1 g/L culture) (Additional file 1: Figure S8) and purified from the soluble cell fraction by IMAC. This soluble fraction was significantly viscous, and an imidazole gradient should be used for ZapB homogeneous purification (Additional file 1: Figure S9). This unusual viscosity already suggested a certain degree of ZapB self-assembly, which was further corroborated by transmission electron microscopy (TEM) analysis of the purified protein. Abundant long protein nanofibers 20 ± 5 nm in width and displaying a regular striated pattern were observed (Fig. 3a), in excellent agreement with the nanostructures visualized in a previous study [42].

We analyzed the secondary structure content of self-assembled ZapB by circular dichroism (CD) spectroscopy. The spectrum is characteristic of an α-helical structure, with two minima at 208 nm and 222 nm (Fig. 3b). Some studies have revealed that the 222/208 nm ratio allows discriminating individual α-helices from coiled-coils, owing to the different periodicity of the two folds. A 222/208 nm ratio ≥ 1 is indicative of coiled-coil structures, and ratios ≤ 0.86 can be attributed to individual α-helices in a protein structure [72, 73]. For ZapB, the 222/208 nm ratio is 0.99, indicating that, as anticipated by the protein crystal structure (PDB: 2JEE), ZapB keeps the coiled-coil fold in the macromolecular fibrillar assembly. The absence of a β-signature in the CD spectrum of self-assembled ZapB is consistent with a non-amyloid nature. This trait was confirmed using the Congo Red (CR) and Thioflavin-T (Th-T) amyloid dyes. Both analyses indicated that the ZapB nanofibers do not have an amyloid-like nature, since the spectra of both dyes in the presence and absence of the protein (buffer alone) are fairly similar (Fig. 3c, d).

### ZapB IBs exhibit a coiled-coil conformation

In order to explore whether, like the nanofilaments purified from the soluble cell fraction, ZapB IBs are sustained by coiled-coil interactions, they were purified from the insoluble cell fraction (Additional file 1: Figure S10) and their secondary structure content analyzed by CD spectroscopy and Fourier Transform Infrared Spectroscopy (FTIR).

**Fig. 4:**
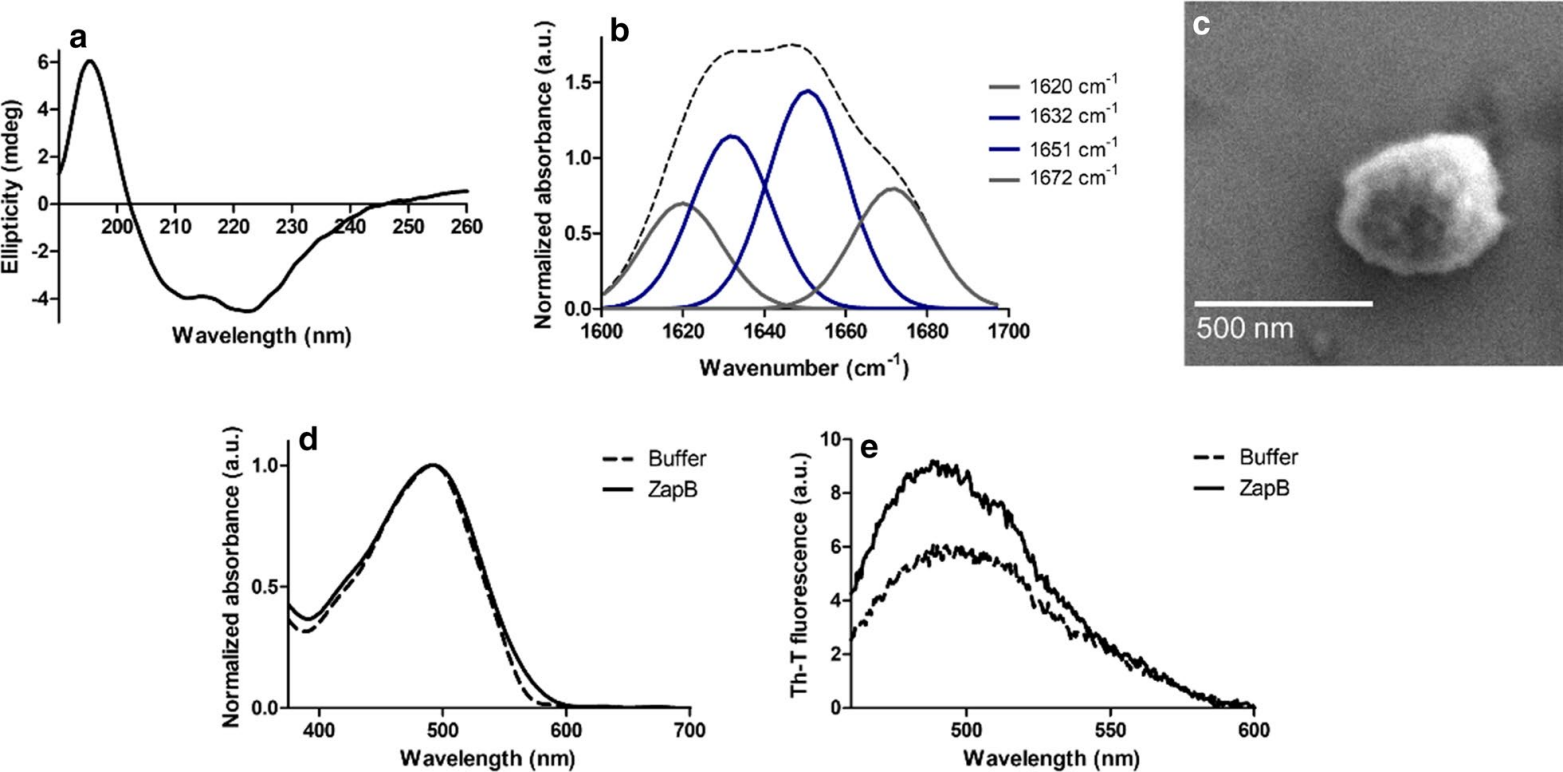
Conformational properties of ZapB IBs. **a** Far-UV circular dichroism spectrum. **b** FTIR absorbance spectrum in the amide I region (dashed line). The different component bands (solid lines) were obtained by deconvolution of the absorbance spectrum. **c** SEM image of a ZapB IB. **d** Congo-Red absorbance spectra. **e** Th-T fluorescence emission spectra

The Far-UV CD spectrum of ZapB IBs resembles the one obtained for the nanofibrillar solution (Fig. 4a). The packing of ZapB into IBs seems to favor the preservation of the coiled-coil conformation since the 222/208 nm ratio of these aggregates is 1.14. We recorded the infrared spectra of ZapB IBs in the amide I region of the spectrum (1700–1600 cm^−1^), corresponding to the absorption of the main chain carbonyl group and sensitive to protein conformation (Fig. 4b). It is important to note here, that in coiled-coils, the supercoil bending of the α-helices results in a spectral splitting of the α-helical IR amide I band and in a shift to lower wavenumbers [74]. FTIR measurements on the model coiled-coil GCN4 demonstrated the assignment of the solvated portion of the coiled-coil to a low helix frequency (1631 cm^−1^); in contrast, the buried helix frequency (1651 cm^−1^) is observed for residues in the interior of the coiled-coil. These two signals alone accounted for the 78% of the GCN4 spectral area [75, 76]. Similarly, the major contributors to ZapB IBs IR spectra are two signals at 1632 cm^−1^ and 1651 cm^−1^ bands, accounting for the 28% and the 35% of the absorbance spectrum area, respectively. Additional bands at 1620 cm^−1^ and 1672 cm^−1^ were detected, the latest one being also present in the IR spectra of GCN4 and other coiled-coil proteins [75]. When observed using Scanning Electron Microscopy (SEM) (Fig. 4c), these IBs displayed a spherical shape. The non-amyloid nature of the IBs was confirmed using both CR and Th-T amyloid dyes, observing similar absorbance and fluorescence spectra for the IBs and the buffer alone, respectively (Fig. 4d, e).

Overall, the CD and FTIR data converge to indicate that ZapB IBs consist mostly of coiled-coil molecules.

### ZapB-GFP and ZapB-mCherry proteins are produced as fluorescent IBs in *E. coli*

**Fig. 5:**
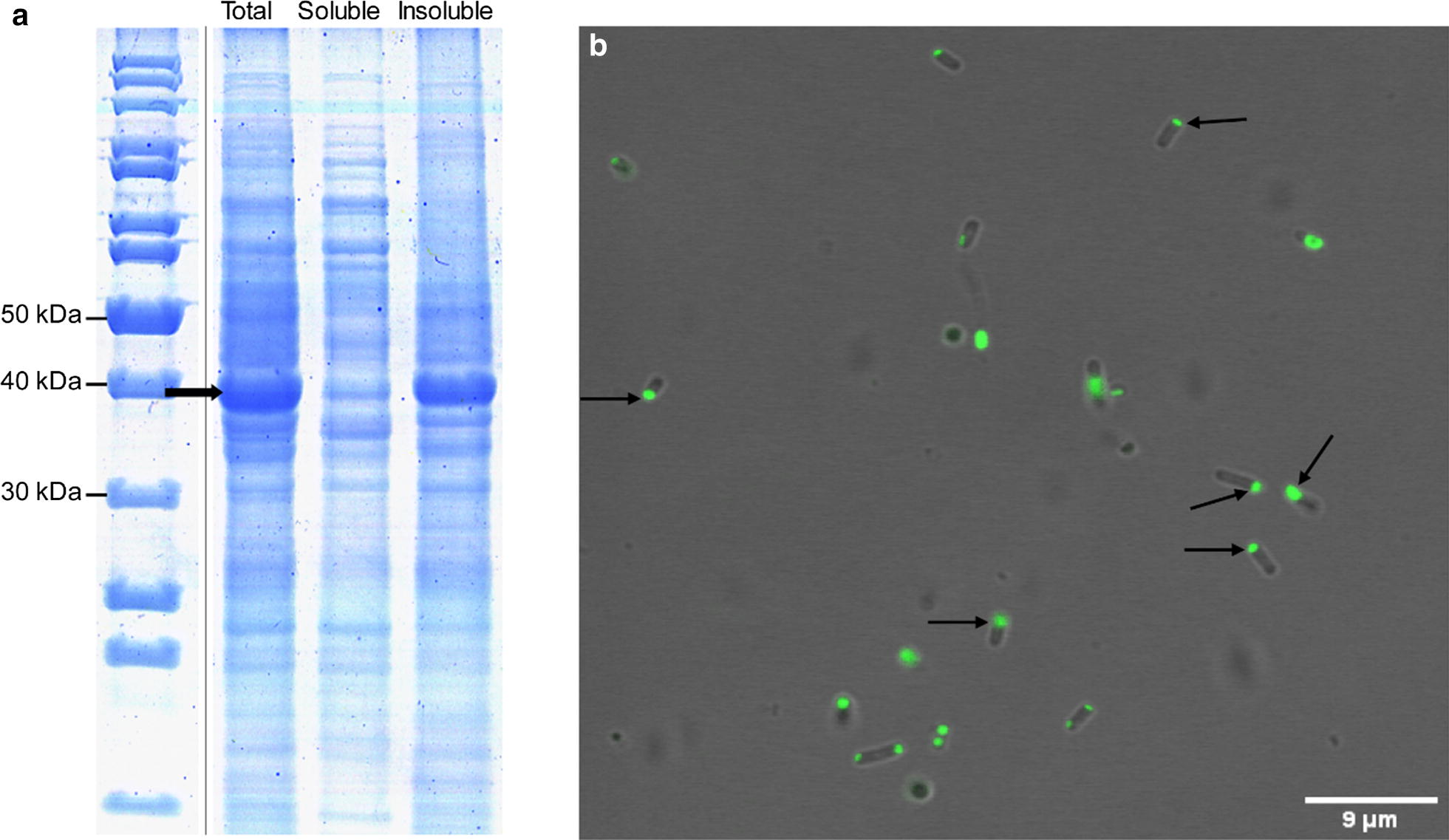
Cellular distribution of ZapB-GFP IBs. **a** SDS-PAGE of intact cells, soluble, and insoluble cellular fractions. The arrow indicates the ZapB-GFP. **b** Visualization of ZapB-GFP IBs in intact *E. coli* cells by confocal microscopy. Some IBs located at the poles of cells are indicated

In order to test the ability of ZapB to assist the formation of functional IBs inside bacteria, ZapB was N-terminally fused to GFP and expressed in *E. coli*. After induction of protein expression, the soluble and the insoluble cellular fractions were separated by centrifugation and analyzed by SDS-PAGE. As shown in Fig. 5a, the ZapB-GFP fusion protein was mainly located in the insoluble fraction (~ 95%), whereas, non-tagged insoluble GFP accounts for ~ 30% of the recombinant protein (Additional file 1: Figure S11). Thus, the fusion of the two polypeptides facilitates GFP deposition. We used fluorescence confocal microscopy to localize the GFP fluorescence emission in *E. coli* intact cells. As expected, the GFP signal was confined mainly in IBs placed at the poles of cells (Fig. 5b).

GFP is considered the default fluorescent protein for most applications, but when dealing with deep in vivo imaging, red-shifted fluorescent proteins are preferred since, at these wavelengths, light absorption by tissues is significantly lower [77], among them mCherry is one of the most used variants [78, 79]. We N-terminally fused ZapB to mCherry, in order to asses if we can obtain red fluorescent IBs with potential in vivo applications.

**Fig. 6:**
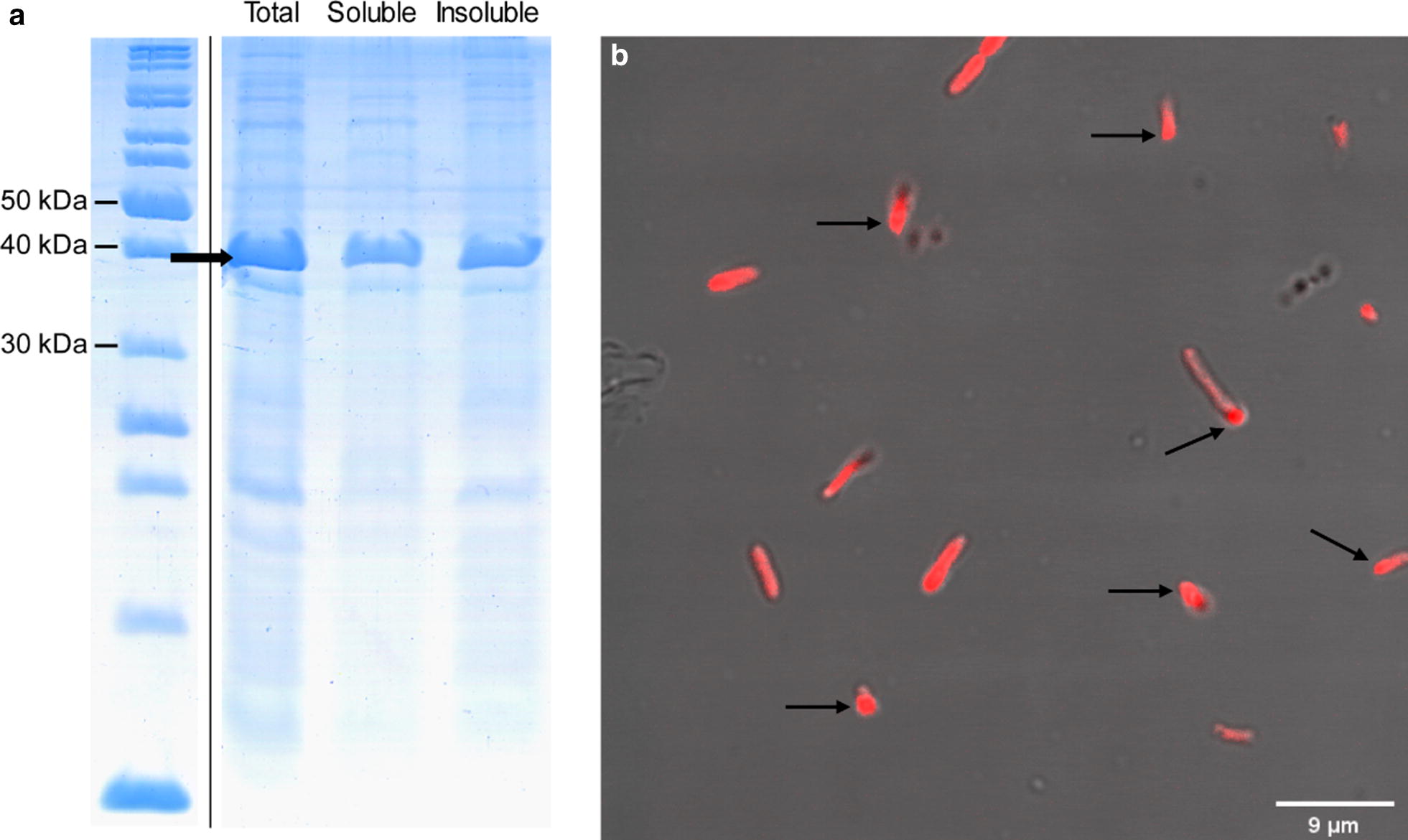
Cellular distribution of ZapB-mCherry IBs. **a** SDS-PAGE of intact cells, soluble, and insoluble cellular fractions. The arrow indicates the ZapB-mCherry. **b** Visualization of ZapB-mCherry IBs in intact *E. coli* cells by confocal microscopy. Some IBs located at the poles of cells are indicated

ZapB-mCherry was expressed in *E. coli*, and the soluble and the insoluble fraction were separated as above. In this case, ~ 55% of the fusion protein is present in the insoluble fraction (Fig. 6a), whereas in non-tagged mCherry only ~ 5% of the protein is insoluble (Additional file 1: Figure S12). The difference between the fraction of ZapB-GFP and ZapB-mCherry located in the respective insoluble fractions likely owes to the highest solubility of the mCherry structure when compared with GFP, as assessed using the AGGRESCAN3D algorithm (Additional file 1: Figure S13). When the location of the red fluorescence was monitored using confocal microscopy, highly fluorescent IBs become evident at the poles. However, their discretization was more difficult than in the case of ZapB-GFP, due to the soluble fusion protein fluorescent background (Fig. 6b).

### GFP and mCherry maintain native spectral properties in ZapB-based IBs

**Fig. 7:**
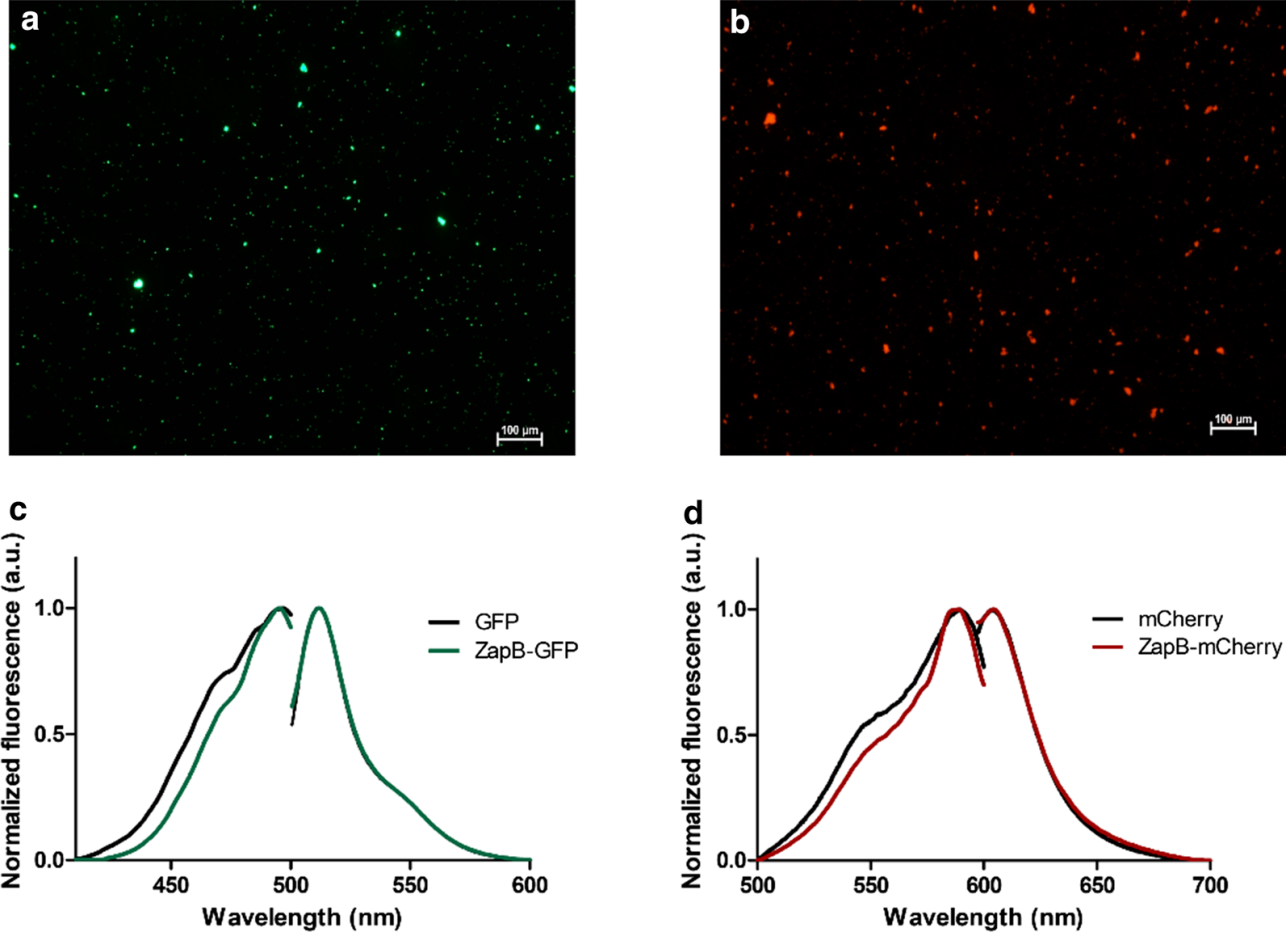
Epifluorescence microscopy images and spectral properties of ZapB-GFP and ZapB-mCherry IBs. **a** Fluorescence microscopy image of purified ZapB-GFP IBs. **b** Fluorescence microscopy image of purified ZapB-mCherry IBs. **c** Excitation and emission spectra of ZapB-GFP IBs and soluble GFP. **d** Excitation and emission spectra of ZapB-mCherry IBs and soluble mCherry

In order to evaluate the impact of the coiled-coil structure in the functionality of the attached fluorescent proteins when embedded in the IBs, we purified both IBs from the insoluble fraction (Additional file 1: Figures S14, S15). The analysis of the purified protein aggregates using an epifluorescence microscope and adequate filters allowed us to observe the presence of abundant green and red fluorescent particles for ZapB-GFP and ZapB-mCherry, respectively (Fig. 7a,b). We compared the spectral properties of the fluorescent proteins trapped in the IBs with those of their soluble and non-tagged counterparts. As it is shown in Fig. 7c, soluble GFP and ZapB-GFP IBs presented identical excitation and emission maxima at 495–496 and 512 nm, respectively. The same behavior was observed when comparing soluble mCherry with ZapB-mCherry IBs, both displaying excitation and emission maxima at 589 and 604 nm, respectively (Fig. 7d). The emission spectra of ZapB-GFP and ZapB-mCherry overlap perfectly with that of the respective soluble untagged fluorescent proteins, whereas, in both cases, the left side of the excitation spectrum is slightly red-shifted when the protein is located within the IBs, which likely respond to differences in crowding and or mobility between soluble and assembled fluorescent proteins.

Overall, these results allow us to confirm that GFP and mCherry keep their activity and, likely, their native conformation inside ZapB-induced IBs.

### ZapB-GFP inclusion bodies contain a coiled-coil conformation

**Fig. 8:**
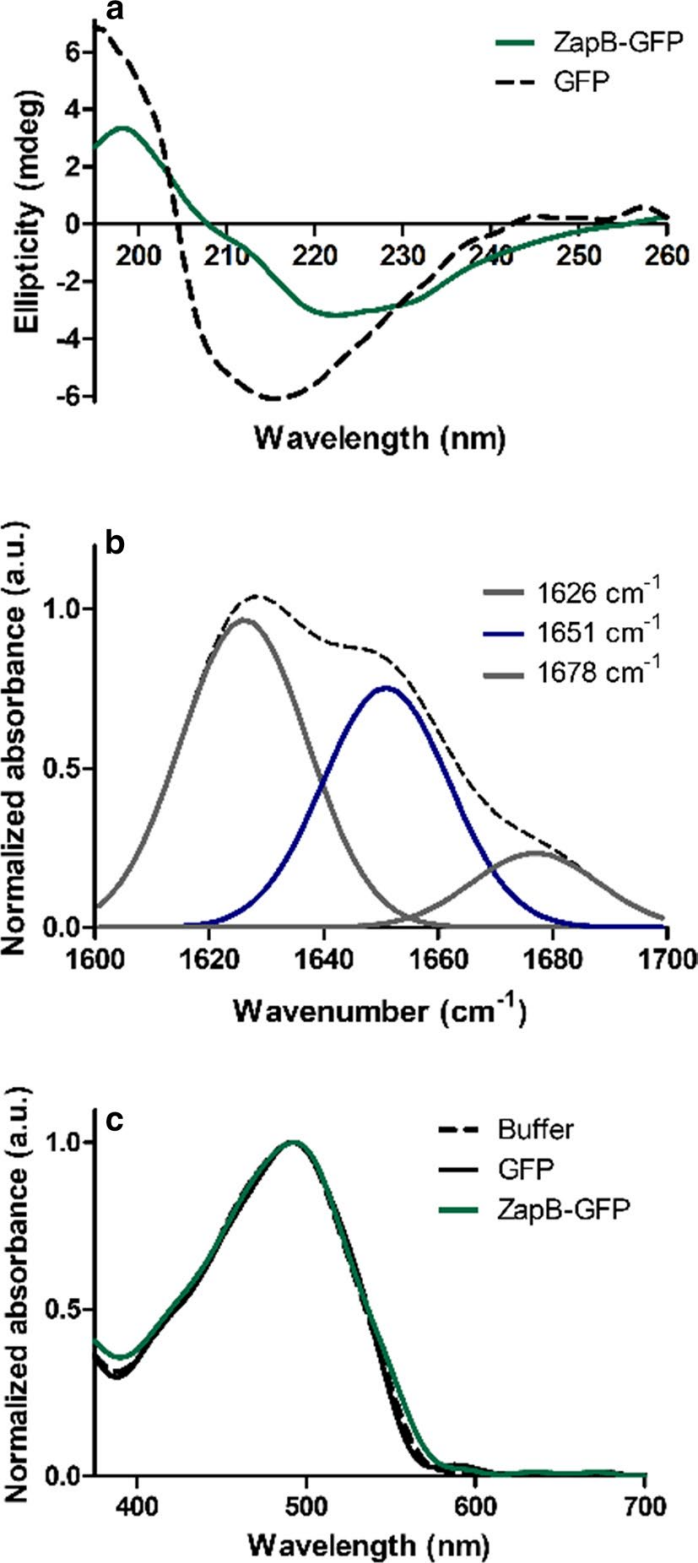
Conformational properties of ZapB-GFP IBs. **a** Far-UV circular dichroism spectrum of ZapB-GFP IBs and soluble GFP. **b** FTIR absorbance spectrum of ZapB-GFP IBs in the amide I region (dashed line). The different component bands (solid lines) were obtained by deconvolution of the spectrum using PeakFit software. **c** Congo-Red absorbance spectra

We selected the ZapB-GFP fusion as a model system to further study the properties of ZapB promoted IBs. We analyzed the secondary structure content of these IBs using CD spectroscopy and FTIR. The far-UV CD spectrum of ZapB-GFP is of course influenced by the all β-sheet structure of GFP (Fig. 8a); still, the two main signatures of α-helices could be detected in the IBs, with the global minimum placed at 222 nm and inflection of the spectrum at 208 nm (Fig. 8a).

When ZapB-GFP IBs were analyzed using FTIR in the amide I region of the spectrum, we could detect, again, the characteristic helical band at 1651 cm^−1^, accounting for 39% of the absorbance area. However, now the major signal is located at 1626 cm^−1^ (49% of the area). This band, likely results from the sum of the low-frequency coiled-coil signal and the β-sheet signal of the fused GFP β-barrel (Fig. 8b). An additional band at 1678 cm^−1^, likely arising from the mixed contribution of the two structurally different domains in the fusion at high frequencies was also evident. Finally, the non-amyloid character of ZapB-GFP IBs was corroborated using the CR amyloid dye, observing similar absorbance spectra in the presence of ZapB-GFP IBs, soluble GFP or only buffer (Fig. 8c).

Overall, the secondary structure analyses of ZapB-GFP IBs suggest that they contain a significant proportion of coiled-coil conformations and do not have an amyloid-like nature. This is likely also the case for ZapB-mCherry IBs, since their binding to Th-T and CR are negligible (Additional file 1: Figure S16).

### Comparison of ZapB-based and amyloid-like IBs

We wanted to compare the properties of the above described coiled-coil-based IBs with those of model amyloid-like IBs. To this aim, we selected fusion of the Alzheimer’s related β-amyloid peptide (Aβ42) and GFP. In previous studies, we have characterized in detail the properties of Aβ42-GFP IBs [22, 80, 81]. The high amyloid propensity of the Aβ42 peptide drives the incorporation of the GFP moiety into β-sheet enriched and fluorescent IBs.

**Fig. 9:**
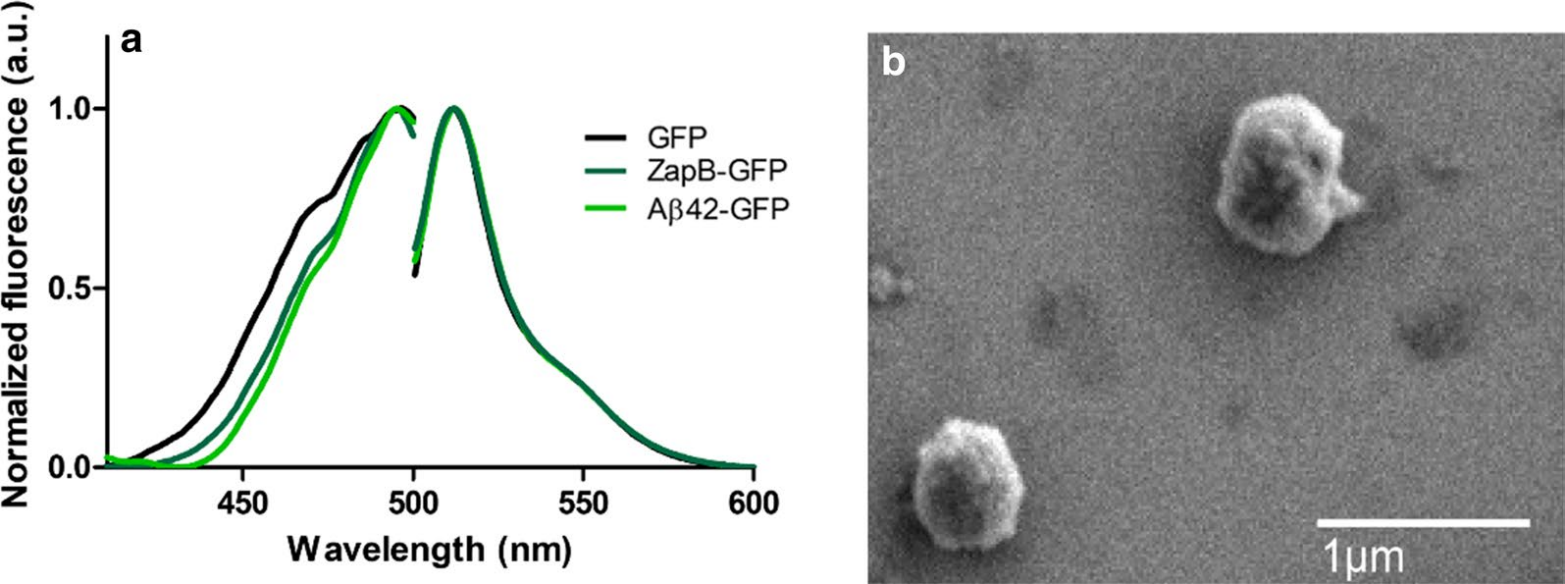
Spectral properties and morphology of ZapB-GFP IBs. **a** GFP-Excitation and emission spectra of ZapB-GFP IBs, Aβ42-GFP IBs, and soluble GFP. **b **SEM image of ZapB-GFP IBs

We expressed Aβ42-GFP (Additional file 1: Figure S17) and purified its IBs (Additional file 1: Figure S18) and compared the spectral properties of GFP in these aggregates with those of ZapB-GFP. Figure 9a demonstrates that Aβ42-GFP and ZapB-GFP IBs share the same excitation and emission spectra, indicating that the active and properly folded GFP they contain is in a similar environmental context. The size of the IBs was analyzed using dynamic light scattering (DLS). Both IBs exhibited similar sizes, with moderately polydisperse distributions and calculated average diameters of 462.2 ± 69.51 nm and 463.3 ± 99.9 nm, for ZapB-GFP and Aβ42-GFP, respectively. The DLS data suggested that ZapB-GFP IBs are quite homogeneous in size (Additional file 1: Figure S19). Effectively, when these aggregates were imaged by Scanning Electron Microscopy (SEM), it was observed that they correspond to submicrometric spherical assemblies (Fig. 9b), and accordingly, they can be assimilated to protein nanoparticles.

**Fig. 10:**
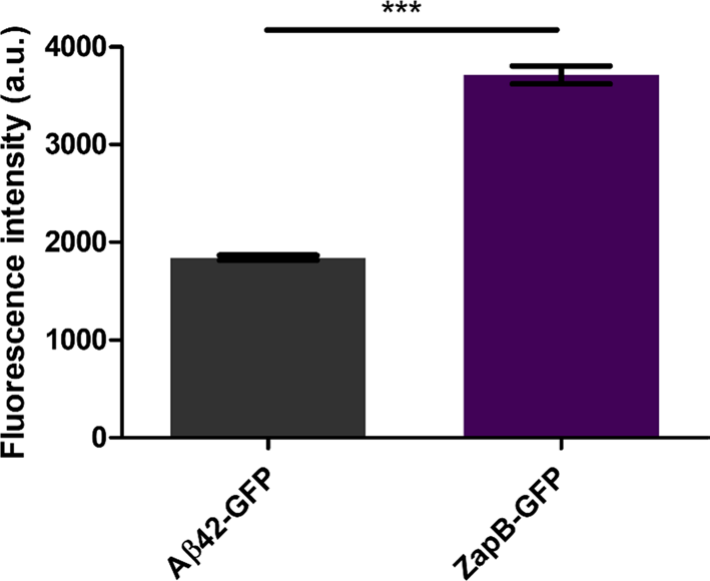
GFP fluorescence of ZapB-GFP and Aβ42-GFP inclusion bodies. Quantification of GFP fluorescence intensity from fluorescence microscopy images using ImageJ. The corresponding intensity of 50 individual fluorescent dots for each sample was analyzed and averaged. The statistical values were derived using the Mann–Whitney Test. A *p* value < 0.001 is indicated as ***. Error bars correspond to SEM

Once confirmed that ZapB-GFP and Aβ42-GFP IBs share spectral properties and dimensions, we wondered if the GFP activity in both nanostructures was similar. To this aim, we analyzed the GFP fluorescence intensity of both IBs by fluorescence microscopy. The mean fluorescence intensity values as obtained from images (Additional file 1: Figure S20) quantification of 50 isolated fluorescent dots in each sample, using ImageJ, revealed that ZapB-GFP IBs exhibited two times higher activity (3713 ± 91.25 a.u.) than Aβ42-GFP IBs (1839 ± 25.23 a.u.) (Fig.  10). This observation is not surprising since the assembly of Aβ42-GFP IBs depends on an aberrant interaction between hydrophobic Aβ42 regions, which leads to a relatively rapid aggregation into amyloid-like structures, with the subsequent inactivation of a least a fraction of the attached globular domains, with their most aggregation-prone sequence stretches contributing to stabilize the aggregate through amyloid-like contacts [22]. Indeed, we have demonstrated that the activities of the IBs formed by 20 Aβ42-GFP variants, bearing different mutations in the Aβ42 moiety, inversely correlate with the aggregation propensities of the peptides [82]. Aβ42-GFP IBs inactivation was favored by increased β-sheet propensity and hydrophobicity and counteracted by increased net charge [54]. ZapB has a negligible β-sheet propensity, is polar, highly charged and has a very low aggregation propensity, compared with Aβ42, all these factors likely contributing to the higher activity of ZapB-GFP IBs. In addition, the inter- and supramolecular assembly of ZapB is directed by native interactions, and not by non-native contacts, as in Aβ42, which are expected to interfere less with the folding and structure of the GFP moiety, and indeed, many coiled–coil domains naturally exist and function appended to globular domains [83].

### ZapB-GFP IBs are innocuous for human cells

**Fig. 11:**
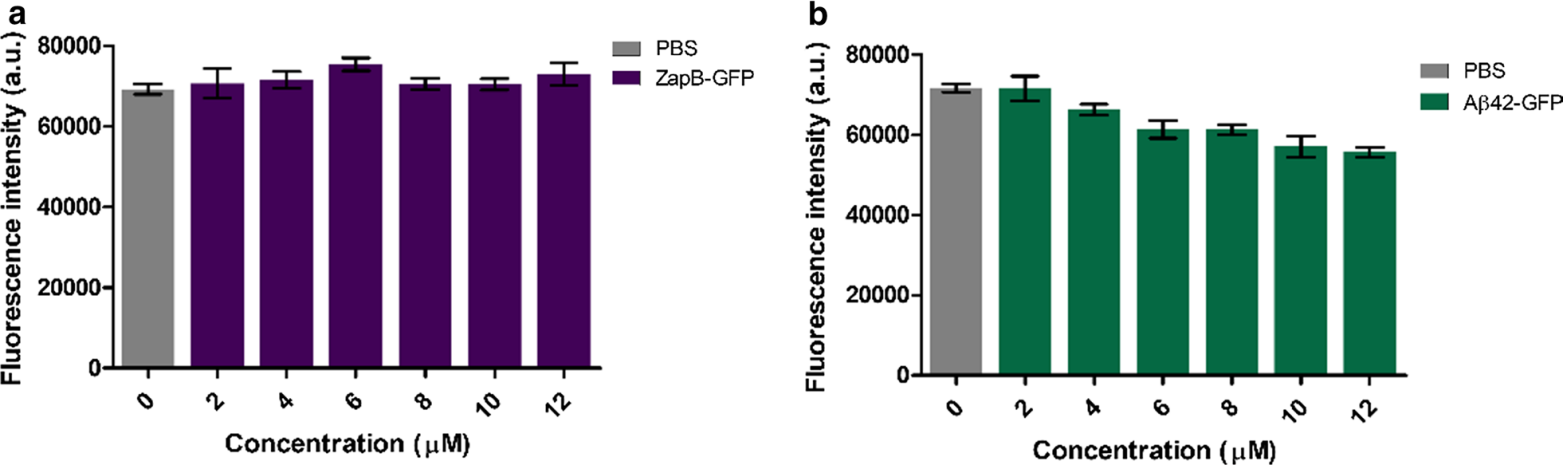
Citotoxicity of ZapB-GFP and Aβ42-GFP IBs. HeLa cells viability was assessed after adding different concentrations (from 2 to 12 µM) of ZapB-GFP **a** and Aβ42-GFP **b** IBs for 72 h. PBS buffer was used as a control

One of the potential advantages of coiled-coil inspired IBs, relative to amyloid-like IBs, is that in the absence of intermolecular β-sheet assemblies that might elicit cytotoxicity, these α-helical-based assemblies should be non-toxic for human cells. To confirm this extent, we incubated HeLa cells with increasing concentrations of ZapB-GFP IBs (from 0 to 12 µM) for 72 h and monitored their viability using PrestoBlue^®^ fluorescent assay. As it can be observed in Fig. 11a, the IBs turned to be innocuous at any of the assayed concentrations, which should make them suitable for in vivo applications. This is contrast with Aβ42-GFP IBs, which exhibit a moderate and concentration-dependent toxicity for HeLa cells (Fig. 11b), in good agreement with the toxicity we described previously for Aβ42 IBs [84].

## Conclusions

IBs have been traditionally regarded as waste reservoirs containing only misfolded and thus non-active and useless proteins. However, it is now clear that at least some proteins can retain certain activity when embedded in these aggregates, turning them into functional sub-micron particles [23, 85]. These nanostructures are finding amazing applications in biotechnology [86] and biomedicine [18, 19].

Conventionally, IBs are enriched in intermolecular β-sheet structure, and contain both amyloid-like and native protein conformations [24]. Unavoidably, a fraction of the target protein becomes inactivated to form the amyloid skeleton that provides mechanical stability to IBs. In order to endorse IBs with tailored functionalities, the particular protein of interest is usually fused to an aggregation-prone polypeptide that act as IB-forming tag [44, 87]. This allows to recruit otherwise soluble proteins into IBs. However, the high-aggregation potential of these tags compromises the folding of the target protein, a fraction of which establishes non-native contacts and becomes an integral part of the amyloid-like architecture, and thus inactive [22].

Because of their amyloidogenic nature, active IBs are assimilated to natural functional amyloids [88]. It is important to note here that, as pathogenic amyloids, functional amyloids found in nature are also inherently cytotoxic [29]; the difference being that nature has evolved dedicated mechanisms to prevent the toxicity of natural assemblies. In the absence of these control mechanisms it cannot can be completely discarded that amyloid-like IBs might contain or release toxic protein conformers.

A way to avoid protein inactivation by non-native interactions and potential toxicity is to run away from non-native-β-sheet based IBs and develop native-α-helix based IBs. We take here a step towards this direction by implementing ZapB based IBs. Catalytic coiled-coil based IBs have been described previously [16, 17, 37–41]. However, our computational analysis suggests that these sequences display lower α-helical and coiled-coil propensities and higher intrinsic aggregation propensities than ZapB, which suggests that a partial transition towards amyloid-like structures upon overexpression cannot be fully discarded, especially because the IBs they promoted were not conformationally characterized.

To avoid the above-mentioned α-helix to β-sheet transition, we have selected ZapB. A natural sequence with extremely high α-helical and coiled-coil propensities, a neglectable intrinsic aggregation propensity and a high polar and anionic character. This coiled-coil protein exhibits the ability to spontaneously assemble into α-helical macromolecular fibrils devoid of any amyloid character. We show how this property can be exploited to obtain innocuous, spherical, relatively homogeneous and highly active sub-micrometric coiled-coil inspired IBs. ZapB expands our existing toolbox to generate immobilized enzymes or biomedical nanocarriers, among other applications. However, the present work constitutes a proof-of-concept, and the ability of ZapB to facilitate the formation of functional IBs when fused to other protein folds should still be demonstrated.

## Methods

### In silico analysis

For the analysis of the coiled-coil tendency, four different predictors were used: Coils, DeepCoil, PCoils and MARCOIL. For the different predictions, the Coiled-coil probability (%) was calculated in order to obtain a quantitative value of the coiled-coil tendency of the analyzed sequences.

Aggregation propensity predictions were performed using Aggrescan and TANGO webservers. In the case of Aggrescan, the Normalized a4v Sequence Sum for 100 residues (Na^4^vSS) was employed as the aggregation propensity value. In the case of TANGO, predictions were made using the default parameters and the *AGG* value was selected as the aggregation propensity value.

For the aggregation propensity predictions considering the 3D structure, AGGRESCAN3D webserver was used with the default parameters and using the following PDBs as an input: PDB: 2JEE for ZapB, PDB: 1FE6 for TDoT and PDB: 3LNR for 3HAMP, PDB: 2Y0G for GFP and PDB: 2H5Q for mCherry. In the case of ZapB and 3HAMP proteins, the dimeric structures were generated with PISA. Protein structures and their surfaces were represented with Pymol (DeLano Scientific, LLC).

The Grand Average of Hydropathicity (GRAVY score) and the net charge at physiological pH were calculated using the ProtParam tool. The net charge per residue (NCPR) was calculated dividing the net charge by the total number of residues.

Finally, secondary structure predictions were performed using GOR and PSIPRED webservers.

### Protein production and purification

ZapB gene fragment (Additional file 1: DNA and amino acid sequences of ZapB protein) was cloned into a pET-21a vector between *NdeI* and *BamHI* restriction sites. The ZapB-GFP and ZapB-mCherry fusion proteins were generated inserting both target proteins after ZapB sequence, using a linker (SIPGA) and *BamHI* and *EcoRI* restriction sites.

For the production of soluble and His-tagged ZapB, GFP and mCherry proteins, transformed *E. coli* BL21 (Invitrogen, USA) cells were grown aerobically in Luria Broth (LB) medium supplemented with 100 µg/ml of ampicillin. Protein expression was induced with 1 mM Isopropyl β-d-1-thiogalactopyranoside (IPTG) at 30 °C for 12 h. For protein purification, cells expressing the recombinant protein were harvested by centrifugation (5000*g* for 20 min). After cell lysis by sonication and fractionation, the soluble fraction was collected and injected in a Histrap FF 5 mL column (GE Healthcare, USA) using an ÄKTA (GE Healthcare, USA). After purification, proteins were dialyzed in buffer A (50 mM Tris, 100 mM NaCl, pH 7.4). The purity of these proteins was checked by SDS-PAGE.

For the production of ZapB, ZapB-GFP, ZapB-mCherry and Aβ42-GFP IBs, transformed *E. coli* BL21 (Invitrogen, USA) cells were grown aerobically in LB medium supplemented with 100 µg/mL of ampicillin for ZapB, ZapB-GFP and ZapB-mCherry transformed cells, and with 50 µg/mL of kanamycin for Aβ42-GFP transformed cells. Protein expression was induced with 1 mM IPTG at 30 °C for 12 h. For IBs purification, cells were centrifuged at 5000*g* for 20 min. After that, IBs were purified as described [89]. Briefly, pelleted cells were resuspended in 400 µL of buffer A (50 mM Tris, 100 mM NaCl, pH 7.4) plus 4 µL of 100 mM PMSF and 6 µL of 10 mg/mL lysozyme. After 1 h of incubation at 37 °C, the mixture was cooled in ice and sonicated 3 min at 15% amplitude under 1 s cycles. After that, 4 µL of Nonidet P40 (NP-40) were added and the mixture incubated at 4 °C for 1 h. Then, 10 µL of 1 mg/mL DNase I and 12 µL of 1 M MgSO_4_ were added and the mixture was further incubated at 37 °C for 45 min. IBs were collected by centrifugation at 15.000*g* for 15 min at 4 °C and washed with buffer A (50 mM Tris, 100 mM NaCl, pH 7.4) containing 0.5% Triton X-100. All incubations were done under gentle agitation. Finally, IBs were washed three times with buffer A (50 mM Tris, 100 mM NaCl, pH 7.4) to remove remaining detergent. The purity of the IBs was checked by SDS-PAGE. Protein concentration was estimated measuring the absorbance at 280 nm in a Specord 200 Plus spectrophotometer (Analytik Jena, Germany).

### Circular dichroism (CD) spectroscopy

For the analysis of the secondary structure, ZapB protein, ZapB and ZapB-GFP IBs were exhaustively resuspended in buffer A (50 mM Tris, 100 mM NaCl, pH 7.4). Soluble GFP was diluted in buffer A (50 mM Tris, 100 mM NaCl, pH 7.4) at 5 µM. Sedimentation problems were avoided with thorough resuspension of the sample before the measurements. The correct resuspension of the sample was checked before and after of each measurement. Far-UV CD spectra of the different protein solutions were recorded using a 1 nm bandwith, a response time of 1 s, and a scan speed of 100 nm/min in a Jasco-710 spectropolarimeter (Jasco Corporation, Japan), thermostated at 25 °C. Ten accumulations were averaged for each spectrum.

### Transmission electron microscopy (TEM)

For TEM sample preparation, 10 µL of 10 µM of ZapB protein were deposited onto a carbon-coated copper grid for 10 min and the excess of liquid was removed with filter paper, followed by a negative stain with 10 µL of 2% (w/v) uranyl acetate for 1 min. Grids were exhaustively scanned using a JEM 1400 transmission electron microscope (JEOL Ltd, Japan) operating at 80 kV, and images were acquired with a CCD GATAN ES1000W Erlangshen camera (Gatan Inc., USA). The width of fibers was analyzed using ImageJ, averaging the measures of 5 individual fibers.

### Scanning electron microscopy (SEM) and dynamic light scattering (DLS)

Scanning electron microscopy (SEM) was used in order to analyze the morphology of ZapB and ZapB-GFP IBs. To do that, 10 µL of sample resuspended in water were deposited on silicon wafers (Ted Pella Inc., USA), air-dried and observed using a SEM Merlin (Zeiss Merlin, Germany) operating at 2 kV.

Dynamic light scattering (DLS) was used for a quantitative determination of ZapB-GFP and Aβ42-GFP IBs size. The size of these nanoparticles was determined using a Zetasizer Nano ZS (Malvern Instruments Limited, UK) at 25 °C. Three different measures of ten runs were recorded for each sample.

### Cell fractionation

The distribution of the expressed ZapB, GFP, mCherry and the fusion proteins (ZapB-GFP, ZapB-mCherry and Aβ42-GFP) in *E. coli* cells was analyzed by SDS-PAGE. After protein expression at 30 °C for 12 h, cells were harvested by centrifugation (5000*g* for 20 min) and resuspended in buffer A (50 mM Tris, 100 mM NaCl, pH 7.4). After that, disrupted cells (total fraction) by sonication were centrifuged at 15.000*g* for 15 min at 4 °C, and supernatant (soluble fraction) was separated from pellet (insoluble fraction). The insoluble fraction was resuspended in the same volume of buffer A (50 mM Tris, 100 mM NaCl, pH 7.4) than the soluble fraction and the different fractions were heated at 98 °C for 10 min. After that, 10 µL of each fraction were loaded separately into SDS-PAGE. Band intensity quantification was performed using the ImageJ software in order to estimate the percentage of protein in each fraction.

### Confocal microscopy

*E. coli* BL21 cells expressing ZapB-GFP and ZapB-mCherry proteins at 30 °C for 12 h were centrifuged and resuspended in PBS pH 7.4 to an OD of 0.1. 10 µL of resuspended cells were deposited on top of microscopy poly-l-lysine glass slides, covered with coverslips and observed in a Leica SP5 confocal fluorescence microscope (Leica Microsystems, Germany).

### Epifluorescence microscopy

10 µL of the ZapB-GFP, ZapB-mCherry and Aβ42-GFP IB samples in buffer A (50 mM Tris, 100 mM NaCl, pH 7.4) were deposited on top of microscopy glass slides and covered with coverslips. The fluorescence was observed using an Eclipse Ts2R-FL inverted microscope (Nikon, Japan) using a C-LED470 filter for GFP fluorescence and a C-LED525 for mCherry fluorescence.

To determine the GFP-fluorescence intensity of ZapB-GFP and Aβ42-GFP IBs, images were analyzed using the ImageJ software. 50 fluorescent dots were selected in each image maintaining the same dimensions (height × width) for the different selections. After that, the intensity of these different fluorescent dots was calculated and the average and SEM values were estimated.

### GFP and mCherry fluorescence spectra

Excitation and emission spectra of soluble GFP, ZapB-GFP IBs and Aβ42-GFP IBs in buffer A (50 mM Tris, 100 mM NaCl, pH 7.4) were analyzed in a Jasco FP-8200 fluorescence spectrofluorometer (Jasco Corporation, Japan). Emission spectra were obtained recording the emitted fluorescence between 500 and 600 nm. Excitation spectra were obtained by exciting the samples in a 400–500 nm range.

Excitation and emission spectra of soluble mCherry and ZapB-mCherry IBs in buffer A (50 mM Tris, 100 mM NaCl, pH 7.4) were analyzed using Jasco FP-8200 fluorescence spectrofluorometer (Jasco Corporation, Japan). Emission spectra were obtained recording the emitted fluorescence between 600 and 700 nm. Excitation spectra were obtained by exciting the samples in a 500–600 nm. Three spectra were accumulated at 25 °C with slit widths of 5 nm, a 0.5 nm Interval, and a 1000 nm/min scan rate for each sample.

### Fourier transform infrared spectroscopy (FTIR)

Samples of ZapB and ZapB-GFP IBs were washed with H_2_O to remove the presence of salts. Both samples were placed on the ATR crystal and dried out under N_2_ flow. The experiments were carried out in a Bruker Tensor 27 FTIR (Bruker Optics, USA) supplied with a Specac Golden Gate MKII ATR accessory. Each spectrum consists of 32 acquisitions measured at a resolution of 1 cm^−1^. Data were acquired and normalized using the OPUS MIR Tensor 27 software (Bruker Optics, USA). IR spectrum was fitted employing a nonlinear peak-fitting equation using PeakFit package v4.12 (Systat Software, USA). The area for each Gaussian curve was calculated in the amide I region from 1700 to 1600 cm^−1^ using second derivative deconvolution method in PeakFit package v4.12 (Systat Software, USA).

### Toxicity assay

HeLa cells were cultured in Dulbecco’s Modified Eagle Medium (DMEM) supplemented with 10% Fetal Bovine Serum (FBS) and seeded into 96-well plates. ZapB-GFP and Aβ42-GFP IBs were resuspended in PBS pH 7.4 and added at a range from 2 to 12 µM. For control, the same volume of PBS pH 7.4 was added. Treated and control cells were incubated for 72 h at 37 °C, and then 10 µL of PrestoBlue^®^ reagent (ThermoFisher Scientific, USA) was added and incubated for 10 min. To determine cell viability, fluorescence signal was measured by exciting at 560 nm and collecting at 590 nm in a Victor3 fluorescent plate reader (Perkin Elmer, USA).

### Thioflavin T (Th-T) and congo red (CR) binding

For the Th-T binding assay, ZapB, mCherry. ZapB and ZapB-mCherry IBs, were diluted in buffer A (50 mM Tris, 100 mM NaCl, pH 7.4) and incubated with 25 µM Th-T. Emission fluorescence was recorded using a Jasco FP-8200 spectrofluorometer (Jasco Corporation, Japan) in the 460–600 nm range, using an excitation wavelength of 440 nm and an emission bandwith of 5 nm. The same buffer with 25 μM Th-T and without protein was employed as a control. In the case of ZapB-GFP, Th-T binding assay was not performed due to an overlap between the fluorescence spectra of GFP and Th-T.

For the CR binding assay the different IBs and soluble proteins were diluted in buffer A (50 mM Tris, 100 mM NaCl, pH 7.4) and mixed with CR to a final concentration of 10 µM CR. Optical absorption spectrum was recorded in the range from 375 to 700 nm in a Specord 200 Plus spectrophotometer (Analytik Jena, Germany). Spectrum of protein alone was acquired to subtract protein scattering.

## Supplementary information

**Additional file 1: Figure S1.** Coiled-coil predictions for ZapB. **Figure S2.** Coiled-coil predictions for 3HAMP. **Figure S3.** Coiled-coil predictions for TDoT. **Figure S4.** Secondary structure prediction by PSIPRED server. **Figure S5.** Secondary structure prediction by GOR server. **Figure S6.** AGGRESCAN3D structural aggregation propensity predictions for ZapB, TDoT and 3HAMP. **Figure S7.** Net charge per residue (NCPR) of the different tags. **Figure S8.** SDS-PAGE of the cellular distribution of ZapB. **Figure S9.** SDS-PAGE of ZapB purification by IMAC. **Figure S10.** SDS-PAGE of purified ZapB IBs. **Figure S11.** SDS-PAGE of the cellular distribution of GFP. **Figure S12.** SDS-PAGE of the cellular distribution of mCherry. **Figure S13.** AGGRESCAN3D structural aggregation propensity predictions for GFP and mCherry. **Figure S14.** SDS-PAGE of purified ZapB-GFP IBs. **Figure S15.** SDS-PAGE of purified ZapB-mCherry IBs. **Figure S16.** Characterization of the non-amyloid nature of ZapB-mCherry IBs. **Figure S17.** SDS-PAGE of the cellular distribution of Aβ42-GFP. **Figure S18.** SDS-PAGE of purified Aβ42-GFP IBs. **Figure S19.** DLS spectra of ZapB-GFP and Aβ42-GFP IBs. **Figure S20.** Epifluorescence microscopy images of ZapB-GFP and Aβ42-GFP IBs. DNA and amino acid sequences of ZapB protein.

## Data Availability

All data generated and analyzed during this study are shown in this article and it Additional file 1.
