# Supplementary material for: Coiled-coil inspired functional inclusion bodies
Source: Microb Cell Fact. 2020 Jun 1;19:117. doi: 10.1186/s12934-020-01375-4 (PMC7268670; doi:10.1186/s12934-020-01375-4)
Supplement: Supplementary file 1 — Additional file 1: Figure S1. Coiled-coil predictions for ZapB. Figure S2. Coiled-coil predictions for 3HAMP. Figure S3. Coiled-coil predictions for TDoT. Figure S4. Secondary structure prediction by PSIPRED server. Figure S5. Secondary structure prediction by GOR server. Figure S6. AGGRESCAN3D structural aggregation propensity predictions for ZapB, TDoT and 3HAMP. Figure S7. Net charge per residue (NCPR) of the different tags. Figure S8. SDS-PAGE of the cellular distribution of ZapB. Figure S9. SDS-PAGE of ZapB purification by IMAC. Figure S10. SDS-PAGE of purified ZapB IBs. Figure S11. SDS-PAGE of the cellular distribution of GFP. Figure S12. SDS-PAGE of the cellular distribution of mCherry. Figure S13. AGGRESCAN3D structural aggregation propensity predictions for GFP and mCherry. Figure S14. SDS-PAGE of purified ZapB-GFP IBs. Figure S15. SDS-PAGE of purified ZapB-mCherry IBs. Figure S16. Characterization of the non-amyloid nature of ZapB-mCherry IBs. Figure S17. SDS-PAGE of the cellular distribution of Aβ42-GFP. Figure S18. SDS-PAGE of purified Aβ42-GFP IBs. Figure S19. DLS spectra of ZapB-GFP and Aβ42-GFP IBs. Figure S20. Epifluorescence microscopy images of ZapB-GFP and Aβ42-GFP IBs. DNA and amino acid sequences of ZapB protein. [file 12934_2020_1375_MOESM1_ESM.pdf]

Additional File 1:

Coiled-coil inspired functional inclusion bodies

Marcos Gil-Garcia[1], Susanna Navarro[1] and Salvador Ventura[1]*

[1] Institut de Biotecnologia i de Biomedicina and Departament de Bioquímica i Biologia Molecular, Universitat Autònoma de Barcelona, Bellaterra (Barcelona) 08193, Spain

*Correspondence: salvador.ventura@uab.cat

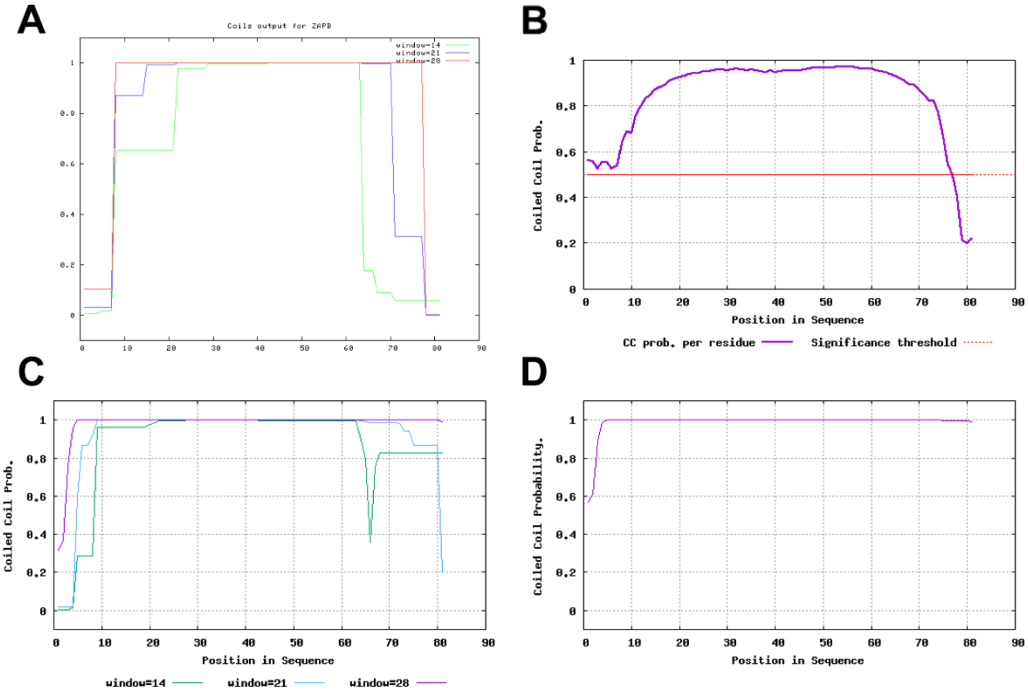

Figure S1. Coiled-coil predictions for ZapB. Coiled-coil predictions for ZapB obtained from four different servers: A) COILS, B) DeepCoil, C) PCoils and D) MARCOIL.

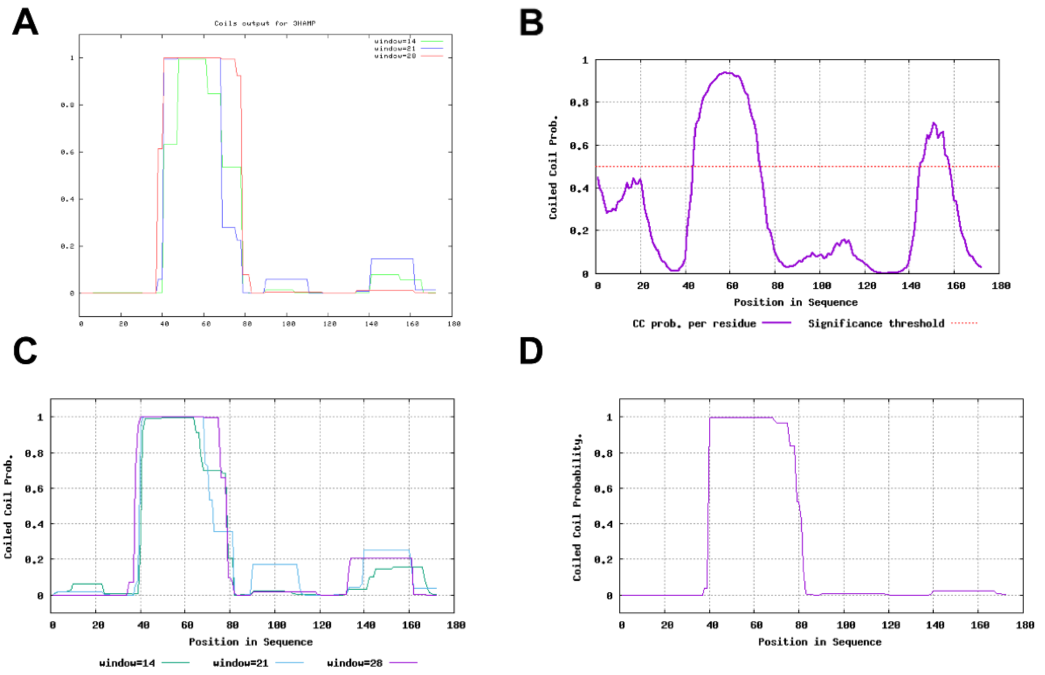

Figure S2. Coiled-coil predictions for 3HAMP. Coiled-coil predictions for 3HAMP obtained from four different servers: A) COILS, B) DeepCoil, C) PCoils and D) MARCOIL.

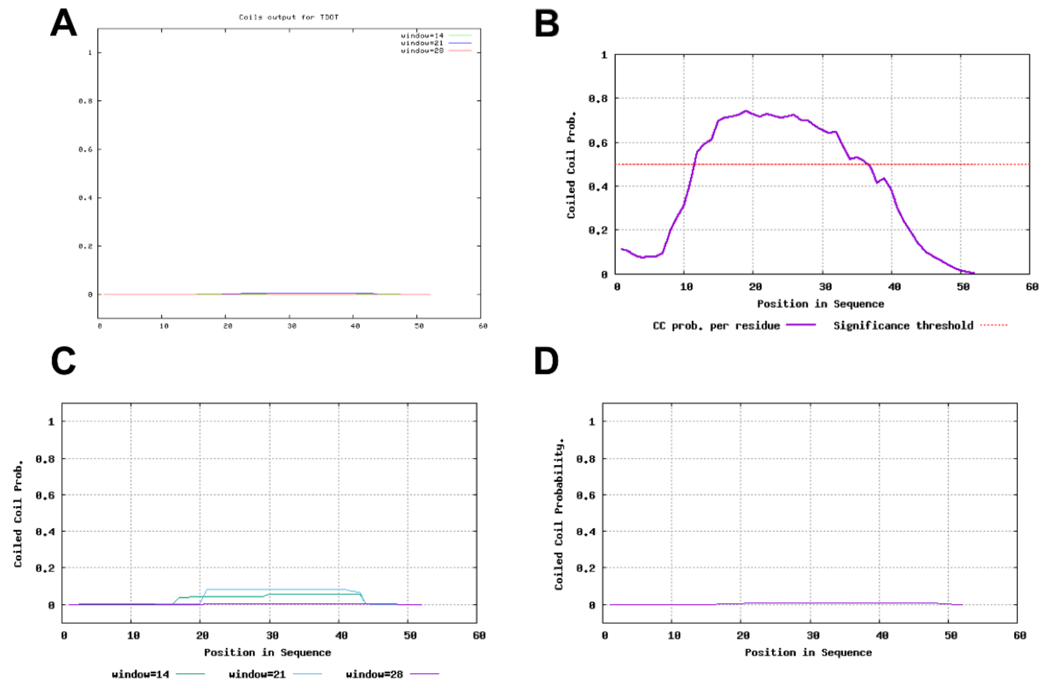

Figure S3. Coiled-coil predictions for TDoT. Coiled-coil predictions for TDoT obtained from four different servers: A) COILS, B) DeepCoil, C) PCoils and D) MARCOIL.

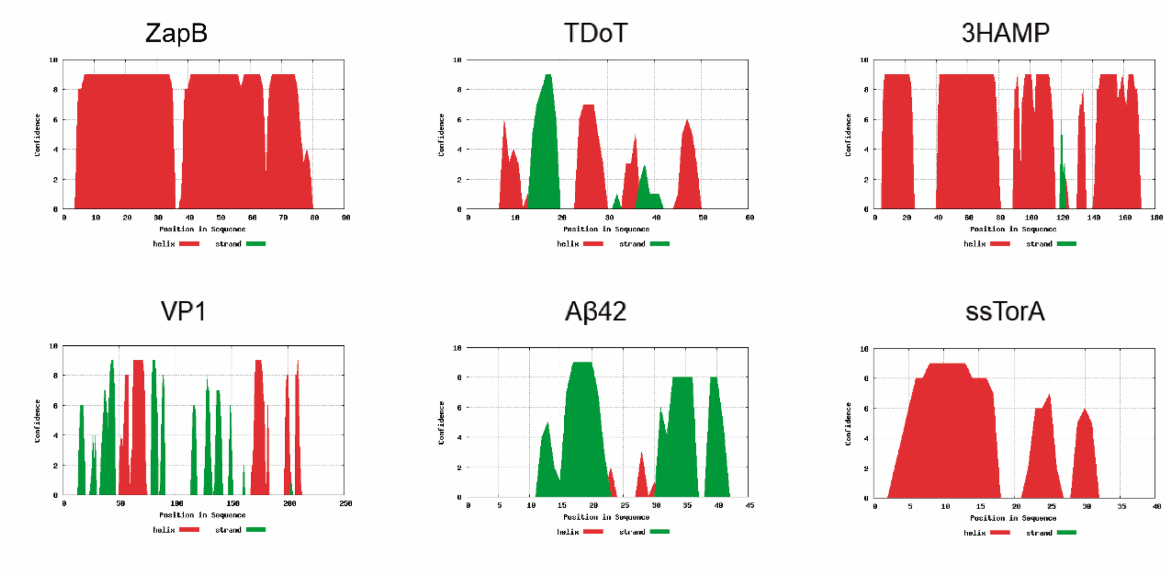

Figure S4. Secondary structure prediction by PSIPRED server. Secondary structure prediction for the six IB-tags by PSIPRED server.

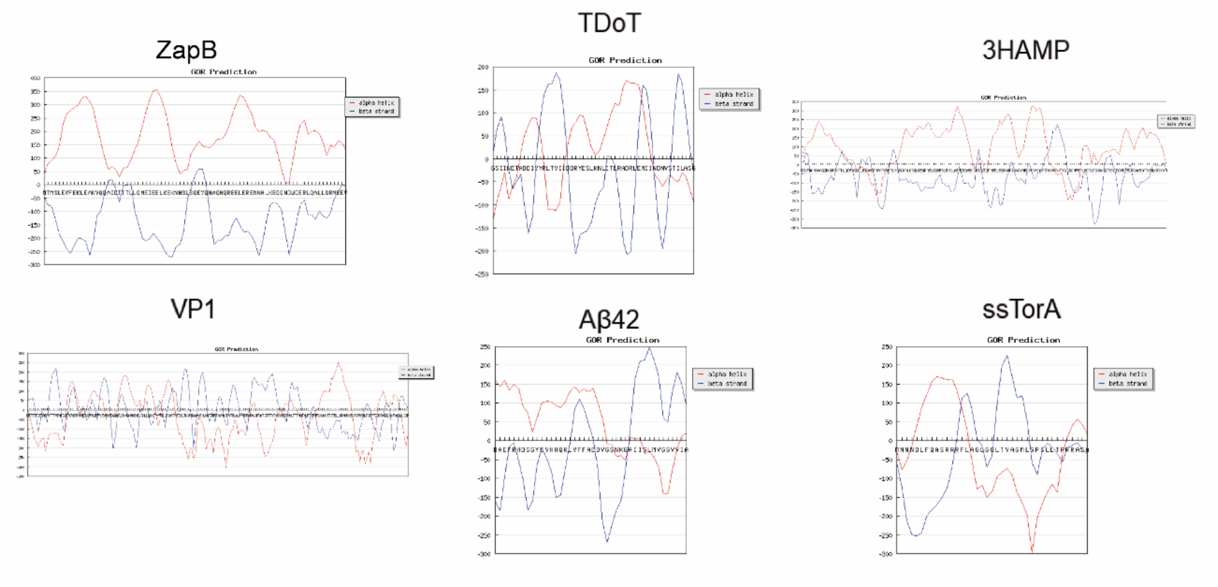

Figure S5. Secondary structure prediction by GOR server. Secondary structure prediction for the six IB-tags by GOR server.

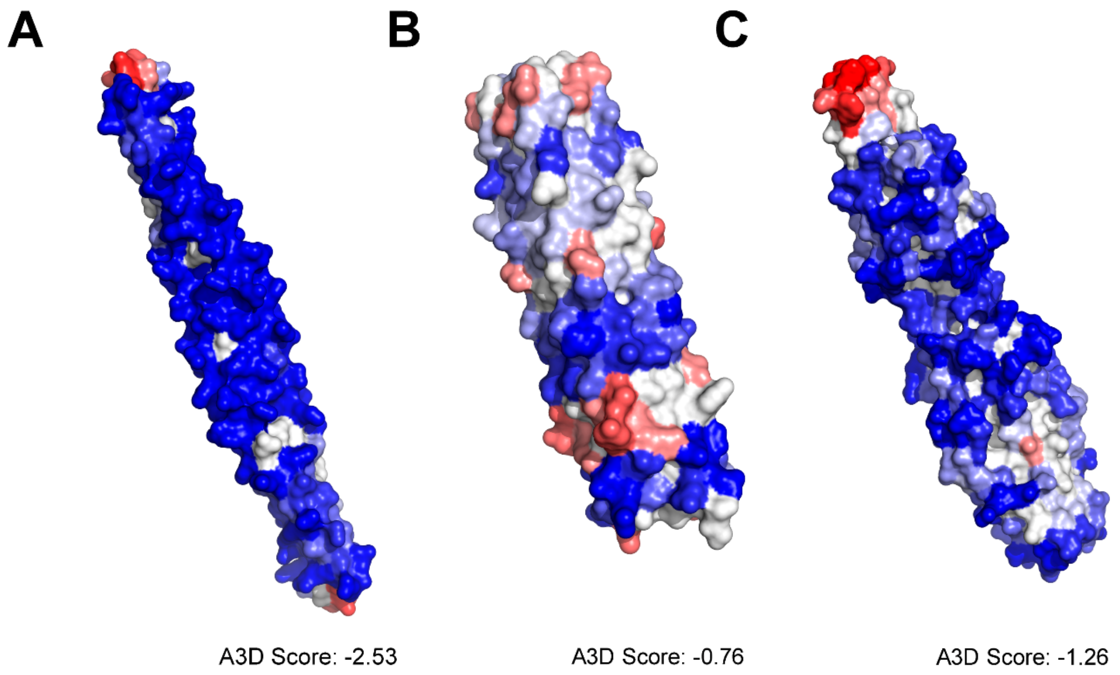

Figure S6. AGGRESCAN3D structural aggregation propensity predictions for ZapB (A), TDoT (B) and 3HAMP (C). The protein surface is colored according to the A3D score in a gradient from blue (high-predicted solubility) to white (negligible impact on protein aggregation) to red (high-predicted aggregation propensity). The A3D Score value for each structure is indicated under the structures. More negative A3D Score values indicate higher solubility.

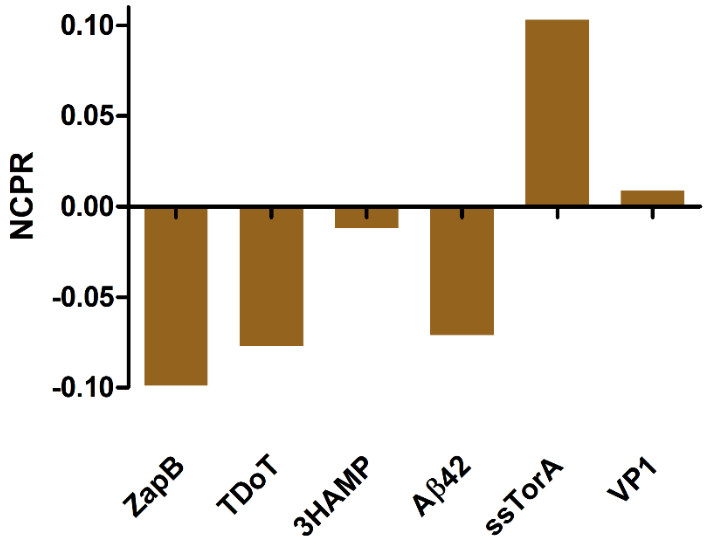

Figure S7. Net charge per residue (NCPR) of the different tags.

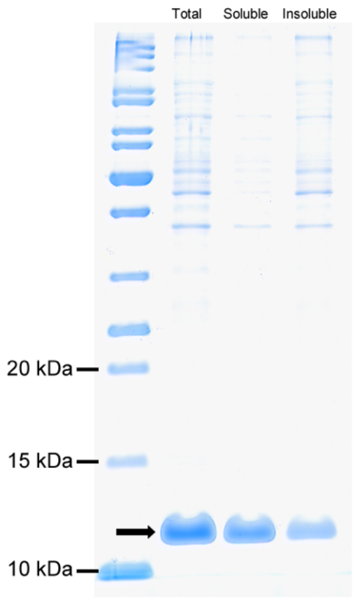

Figure S8. SDS-PAGE of the cellular distribution of ZapB.

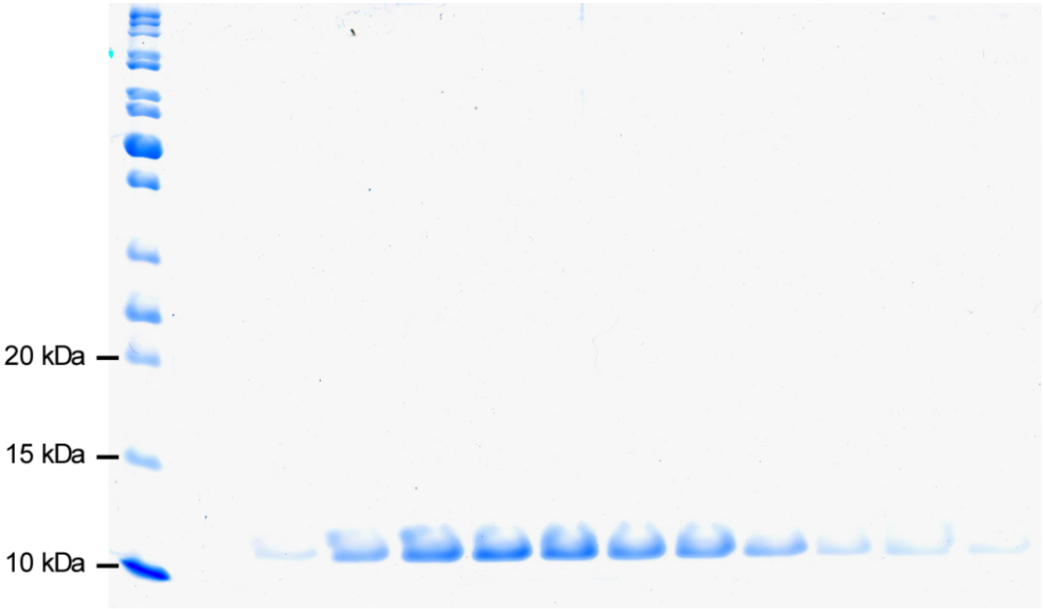

Figure S9. SDS-PAGE of ZapB purification by IMAC. Different elution fractions of ZapB IMAC purification.

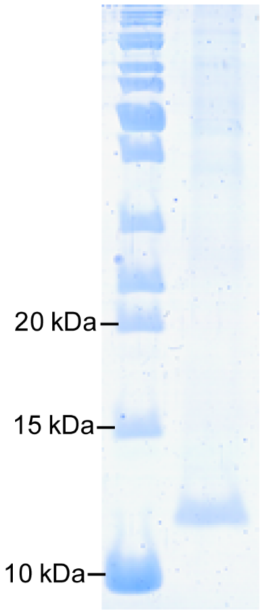

Figure S10. SDS-PAGE of purified ZapB IBs.

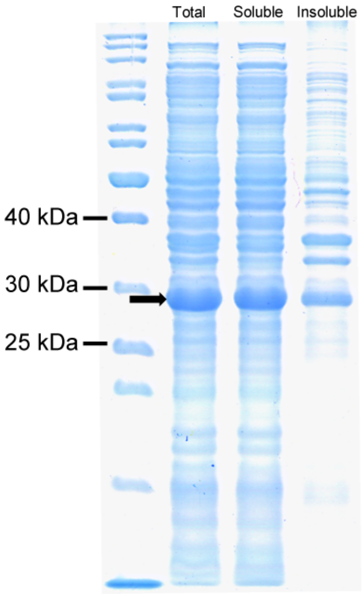

Figure S11. SDS-PAGE of the cellular distribution of GFP.

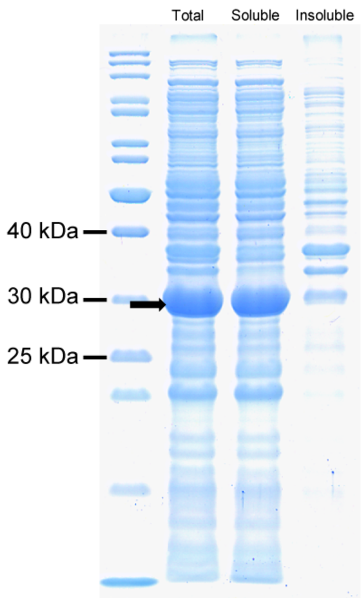

Figure S12. SDS-PAGE of the cellular distribution of mCherry.

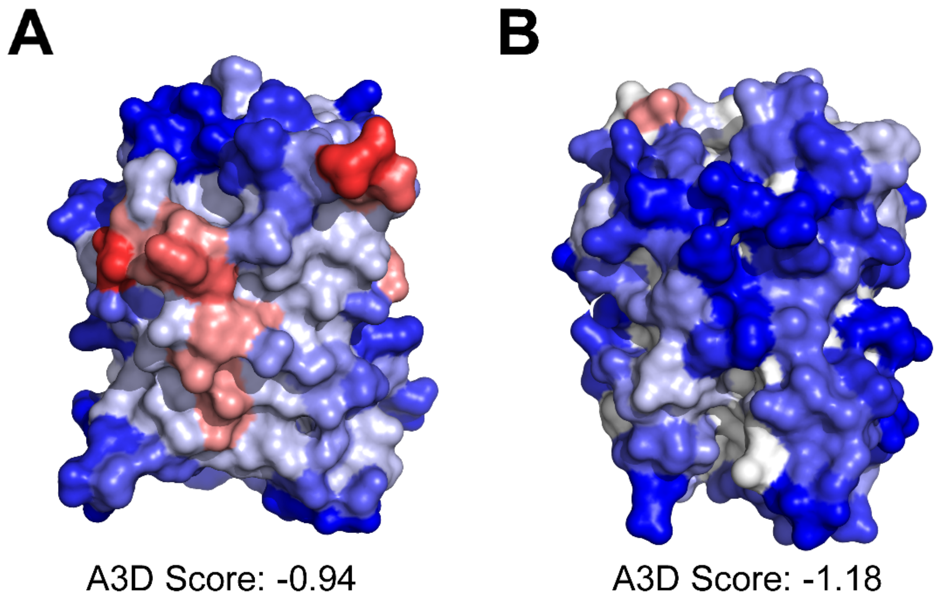

Figure S13. AGGRESCAN3D structural aggregation propensity predictions for GFP (A) and mCherry (B). The protein surface is colored according to the A3D score in a gradient from blue (high-predicted solubility) to white (negligible impact on protein aggregation) to red (high-predicted aggregation propensity). The A3D Score value for each structure is indicated under the structures. More negative A3D Score values indicate higher solubility.

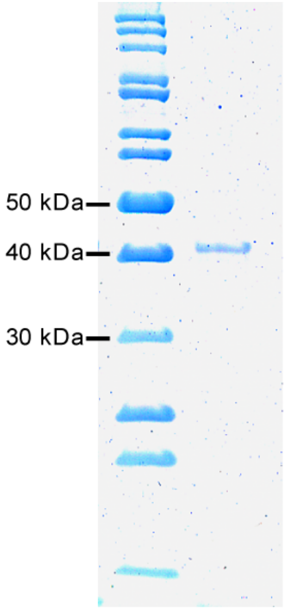

Figure S14. SDS-PAGE of purified ZapB-GFP IBs.

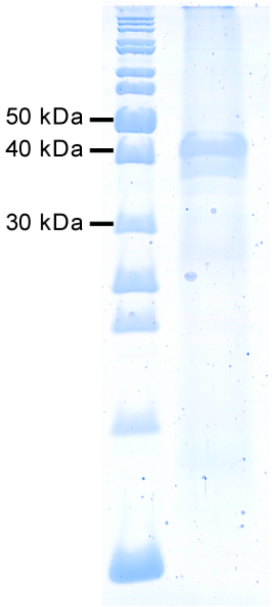

Figure S15. SDS-PAGE of purified ZapB-mCherry IBs.

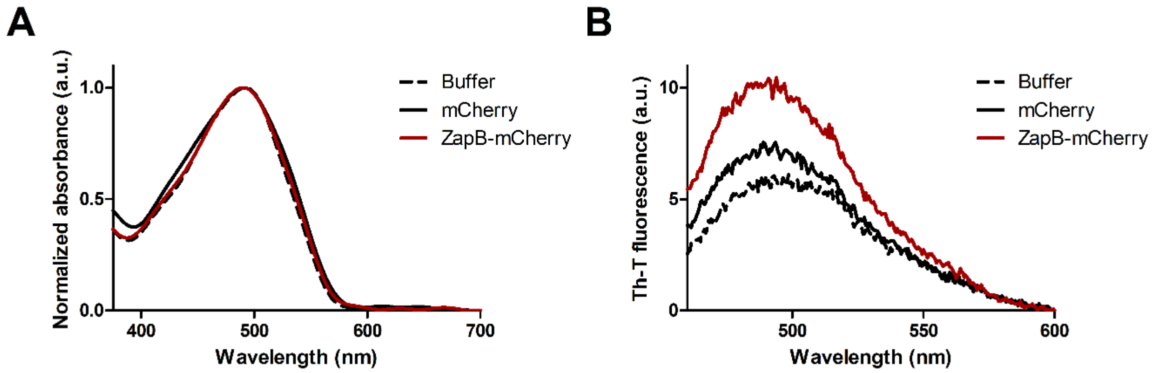

Figure S16. Characterization of the non-amyloid nature of ZapB-mCherry IBs. A) Congo-Red absorbance spectra. B) Th-T fluorescence emission spectra.

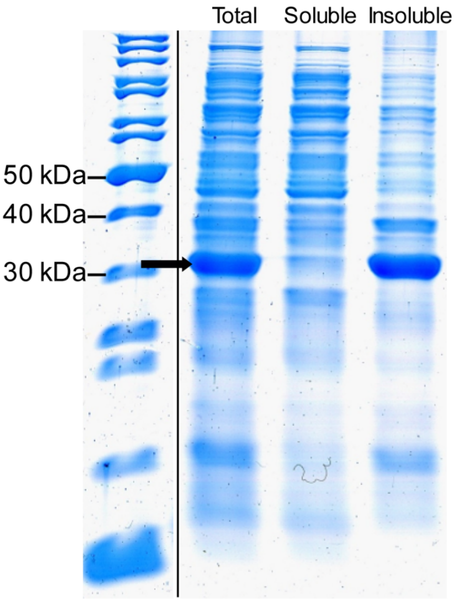

Figure S17. SDS-PAGE of the cellular distribution of Aβ42-GFP.

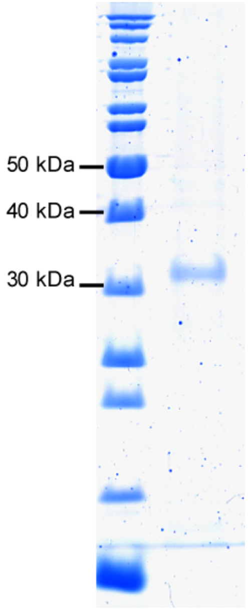

Figure S18. SDS-PAGE of purified Aβ42-GFP IBs.

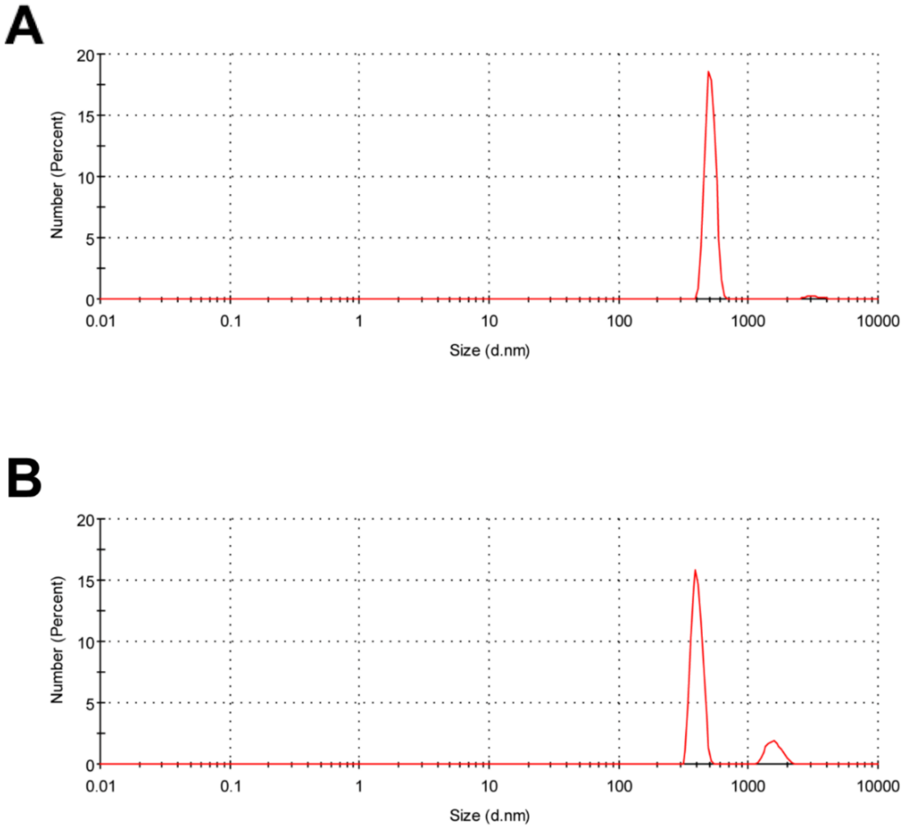

Figure S19. DLS spectra of ZapB-GFP (A) and Aβ42-GFP (B) IBs.

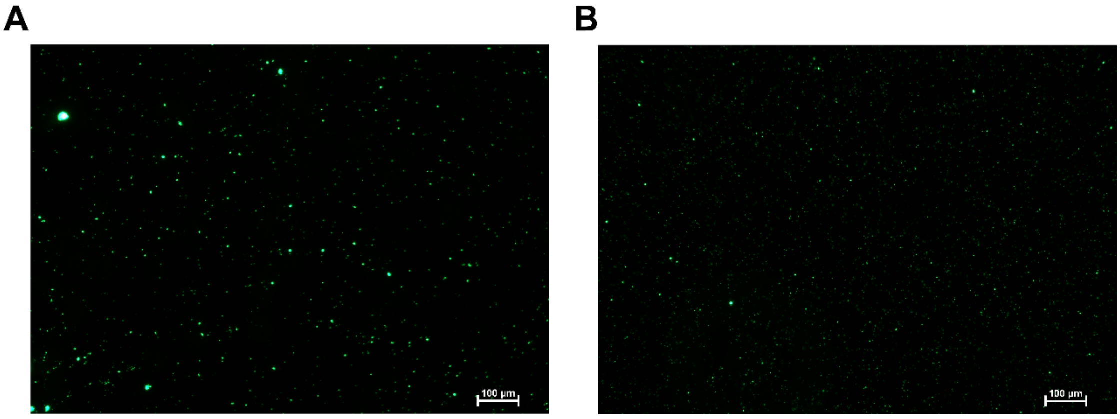

Figure S20. Epifluorescence microscopy images of ZapB-GFP (A) and Aβ42-GFP (B) IBs. A) GFP fluorescence of ZapB-GFP IBs. B) GFP fluorescence of Aβ42-GFP IBs.

DNA and amino acid sequences of ZapB protein

ZapB DNA sequence:

ATGACAATGTCATTAGAAGTGTTTGAGAAACTGGAAGCAAAAGTACAGCAGGCGATTGATACCATCAC

TCTGTTGCAGATGGAAATCGAAGAGCTGAAAGAAAAAAACAACTCACTGTCGCAGGAAGTTCAAAATG

CCCAGCATCAGCGCGAAGAGCTGGAGCGTGAGAACAACCATCTGAAAGAACAGCAGAACGGCTGGCA

GGAACGTCTGCAGGCCCTGCTGGGTCGCATGGAAGAGGTC

ZapB amino acid sequence:

MTMSLEVFEKLEAKVQQAIDTITLLQMEIEELKEKNNSLSQEVQNAQHQREELERENNHLKEQQNGWQER

LQALLGRMEEV
